# The centriculum, a membrane that surrounds *C. elegans* centrosomes, acts as a microtubule filter

**DOI:** 10.1101/2025.08.16.670680

**Published:** 2025-08-18

**Authors:** Richa Maheshwari, Mohammad M Rahman, Abigail Ruddick, Seth Drey, Orna Cohen-Fix

**Affiliations:** The Laboratory of Biochemistry and Genetics, National Institute of Diabetes and Digestive and Kidney Disease, National Institutes of Health, Bethesda MD, 20892, USA

**Keywords:** Centrosome, microtubules, centriculum, *C. elegans*, SPD-5

## Abstract

Centrosomes are the main microtubule-nucleating structures in dividing cells. They are considered membrane-less organelles, but in several cell types they are surrounded by ER-derived membrane. In *C. elegans* early embryos, this membrane forms a dense membrane reticulum, named the centriculum, which was shown to affect centrosome structure and consequently microtubule nucleating capacity. The centriculum is adjacent to the centrosome’s pericentriolar material (PCM) and to abundant, short pericentrosomal microtubules that surround the centrosome. Here we show that the centriculum serves as a microtubule filter, preventing the extension of a fraction of microtubules due to their collision with the centriculum. Changing microtubule number or stability results in a corresponding change in centriculum size, independent of the PCM, indicative of centriculum-microtubule interactions. Moreover, the porosity of the centriculum correlates with the density of microtubules extending beyond the pericentrosomal region, consistent with the centriculum acting as a microtubule filter. Finally, if microtubule-centriculum collisions result in microtubule catastrophe, the filter function of the centriculum could also explain the high concentration of soluble tubulin at the *C. elegans* centrosome.

## Introduction

Centrosomes are cytoplasmic structures that nucleate microtubules, polymers made of α- and β-tubulin heterodimers that form linear, side-by-side, protofilaments, creating a hollow microtubule tube (reviewed in (Aljiboury & Hehnly, 2023)). Microtubules are polar; centrosome-nucleated microtubules have their minus (α-tubulin facing) ends embedded in the centrosome, and their plus (β-tubulin facing) ends extending away from the centrosome. Microtubules are dynamic structures that grow and shrink mainly at their plus end in a manner that is dependent on soluble tubulin concentrations and aided by microtubule associated proteins, such as motor proteins, microtubule polymerases and severing proteins, and more (reviewed in (Gudimchuk & McIntosh, 2021). In dividing cells, centrosomes nucleate microtubules that either form the mitotic spindle or extend to the cell cortex in the form of astral microtubules (reviewed in (Hoffmann, 2021)).

At the core of centrosomes are two centrioles that are surrounded by a pericentriolar material (PCM), a proteinaceous structure whose properties are just beginning to emerge (reviewed in (Magescas et al, 2019; Pimenta-Marques & Bettencourt-Dias, 2020; Woodruff et al, 2014). The PCM is rich in coiled-coil proteins that form a lattice, or scaffold, onto which additional PCM proteins are recruited. One of the main components of the PCM in *C. elegans* is SPD-5 (Hamill et al, 2002), which is functionally homologous to Centrosomin/Cnn in *Drosophila* (Megraw et al, 2001; Megraw et al, 1999; Timothy et al, 1999) and CDK5RAP2 in vertebrates (Fong et al, 2008). The PCM also includes γ-tubulin (TBG-1 in *C. elegans*), which, along with associated proteins that form the γ-tubulin ring complex (γ -TuRC), serves to nucleate microtubules ((Moritz et al, 1995; Zheng et al, 1995) reviewed in (Gao et al, 2024)). At the onset of mitosis, the PCM increases in size in a process known as centrosome maturation ((Khodjakov & Rieder, 1999) reviewed in (Vasquez-Limeta & Loncarek, 2021)). The expansion of the *C. elegans* PCM, as measured by SPD-5 accumulation, involves the incorporation of soluble SPD-5 throughout the volume of the SPD-5 lattice (Laos et al, 2015; Woodruff et al, 2015). This process is facilitated by phosphorylation of SPD-5 by the polo-like kinase PLK-1 (Cabral et al, 2019; Nakajo et al, 2022; Rios et al, 2024; Woodruff et al., 2015; Wueseke et al, 2016). PLK-1 phosphorylation of SPD-5 is independently required to promote γ-tubulin recruitment to the PCM (Ohta et al, 2021). The role of PLK-1 in centrosome maturation is conserved (Conduit et al, 2015; Lane & Nigg, 1996; Lee & Rhee, 2011).

Centrosomes are considered membrane-less organelles, although there are numerous examples of ER-derived membrane accumulation adjacent to the centrosome (Araújo et al, 2023; Bergman et al, 2015; Bobinnec et al, 2003; Diaz et al, 2019; Harris, 1975; Karabasheva & Smyth, 2019; Rollins & Blankenship, 2023; Schlaitz, 2014; Terasaki, 2000; Waterman-Storer et al, 1993). This peri-centrosomal membrane was analyzed in the 1-cell *C. elegans* embryo by volume electron microscopy at 9 nm isotropic resolution and found to be made of a dense membrane reticulum, leading to its name the centriculum (Maheshwari et al, 2023). While the structure of peri-centrosomal membranes in other organisms has yet to be examined at this resolution, the presence of the ER curvature-inducing proteins Rtn1 and ReepB at peri-centrosomal membranes of *Drosophila* embryos (Diaz et al., 2019) suggests a similar structure. In *C. elegans*, downregulation of atlastin, which forms ER-ER junctions (Hu & Rapoport, 2016), led to an increase in centriculum size (Maheshwari et al., 2023). This was accompanied by an increase in both centrosome size and the amount of PCM material (as measured by the area and total amount of fluorescently tagged PCM proteins), and increased microtubule-nucleating capacity (Maheshwari et al., 2023). Likewise, in *Drosophila* embryos, affecting ER structure, including prominent alterations to the membrane surrounding centrosomes, caused defects in centrosome and spindle structure (Araújo et al., 2023; Rollins & Blankenship, 2023). Thus, at least in these systems, centrosome function is affected by the membrane adjacent to it, indicating that the centrosome may not be as membrane-less as previously assumed.

**Figure 1:**
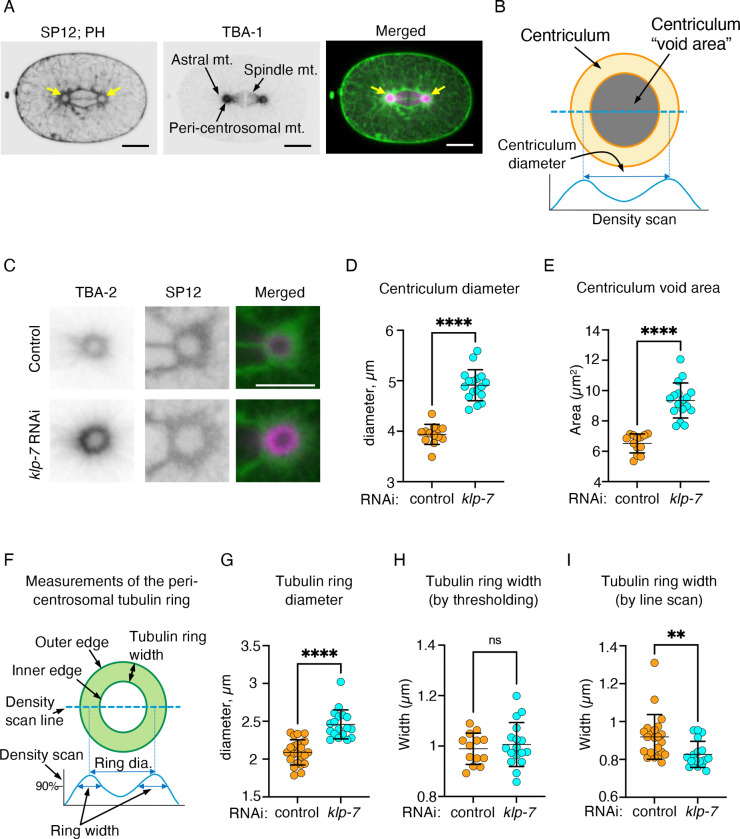
Downregulating KLP-7 increases centriculum size but does not increase the width of the peri-centrosomal tubulin ring. **A)** A *C. elegans* 1-cell embryo (strain OCF193) at metaphase expressing SP12::GFP and PH domain::GFP (ER and plasma membrane markers, respectively; left panel and in green in merged panel) and TBA-1::RFP (microtubules; middle panel and in magenta in merged panel). Three types of microtubules are indicated: astral microtubules, which are nucleated at the centrosome and extend toward the cortex, spindle microtubules, which extend toward the chromosomes (not shown, but are located in the gap between the two half spindles), and peri-centrosomal microtubules, which accumulate in the vicinity of the centrosome but do not extend much past it. The two centricula flaking the spindle are indicated by yellow arrows in the merged image. Scale bar=10 μm. **B)** Schematic representation of the centriculum: The inner and outer edges (dark orange line) of the centriculum (light orange) are shown. The area encompassed by the inner edge of the centriculum is defined as the centriculum “void” (in gray; in vivo, this is where the centrosome resides). The diameter of the centriculum is measured by a density scan of two perpendicular lines that traverse the centriculum and pass through the center of the void (a schematic trace of one line is shown). The distance between the two peaks is measured, and the diameter of the centriculum is the average of the two distances obtained from the perpendicular lines, as described in (Maheshwari et al., 2023). **C)** Representative images of centricula from 1-cell embryos (MSN146) at metaphase expressing mCherry::SP12 (green in the merged images) and GFP::TBA-2 (magenta in the merged images), from worms treated with RNAi against a control (top row) or *klp-7* (bottom row). Scale bar= 5 μm. **D and E)** Quantification as described in panel B of centriculum diameter (D) and centriculum void area (E) in 1-cell embryos from control and *klp-7* RNAi treated worms, as shown in panel C. n=13 and n=17 for the control (orange) and *klp-7* RNAi (cyan), respectively. p<0.0001 using unpaired t test (panel D) or Mann-Whitney test (panel E). Error bars indicate mean and standard deviation. **F)** Schematic representation of the peri-centrosomal “tubulin ring”: in vivo, pericentrosomal microtubules form a sphere around the centrosome (Redemann et al., 2017), which appears as a ring when imaged using fluorescently-tagged tubulin subunits (for example, see panel C). The inner and outer edges (dark green) of the ring (light green) are shown. Tubulin ring diameter was determined by line scanning, as was described in panel (B) for the centriculum. Tubulin ring width is defined as the distance between the inner and outer edges. This was determined by one of two methods: thresholding or line scan. See text and Materials and Methods for detail. **G)** Quantification of tubulin ring diameter in 1-cell embryos from control and *klp-7* RNAi treated worms, as shown in panel C. n=22 and n=18 for the control (orange) and *klp-7* RNAi (cyan), respectively. p<0.0001 using Mann-Whitney test. Error bars indicate mean and standard deviation. **H and I)** Quantification of tubulin ring width using thresholding (H) or the line scan (I) methods under control (orange) or *klp-7* (cyan) RNAi conditions. The width of the tubulin ring was the same under these two conditions using the thresholding method (p=0.5594, unpaired t test) and was significantly smaller following *klp-7* RNAi using the line scan method (p=0.0017, Mann-Whitney test). n=13 and 17 in panel H, and n= 22 and 18 in panel I for control and *klp-7* RNAi, respectively. Error bars indicate mean and standard deviation.

It is well documented that during *C. elegans* early embryogenesis, the majority of microtubules nucleated by the centrosome terminate in the vicinity of the centrosome (for example, see (Albertson, 1984; Redemann et al, 2017) and Fig 1A; defined here as peri-centrosomal microtubules). A similar accumulation of short microtubules around centrosomes is seen in embryos of the fish medaka (Kiyomitsu et al, 2024), *Drosophila* (Rollins & Blankenship, 2023) and sea urchin (Xie et al, 2025). The *C. elegans* centrosome is also able to accumulate soluble tubulin at a concentration that is 10-fold higher than the cytoplasm (Baumgart et al, 2019). The precise mechanism that facilitates this accumulation is not known. A condensate of SPD-5 with additional PCM proteins was shown to concentrate soluble tubulin *in vitro* (Woodruff et al, 2017), although not to the extent observed *in vivo* (Baumgart et al., 2019). The centriculum provides an attractive mechanism for these two phenomena: First, if microtubules, as they elongate, cannot displace centriculum membrane in their path, these microtubules will stop elongating, forming the observed peri-centrosomal microtubules accumulation. In this way, the centriculum would act as a microtubule filter, limiting the number of astral microtubules that can fully elongate, potentially reducing the competition for cytoplasmic tubulin pools. Second, if the terminated microtubules undergo catastrophe, akin to the catastrophe that occurs when microtubules encounter the plasma membrane (Bouvrais et al, 2021), this could also provide a source for the exceedingly high soluble tubulin concentration at the centrosome (Baumgart et al., 2019). We therefore set out to determine whether the centriculum functions as a microtubule filter.

## Results

### Increased centriculum size by partial downregulation of atlastin, but not KLP-7, results in a wider tubulin ring.

Our hypothesis, that the centriculum serves as a microtubule filter, rests on the idea that the membrane reticulum that makes up the centriculum can resist microtubule elongation. We have previously shown, using EM tomography data, that there are microtubules that end at the centriculum membrane (Maheshwari et al., 2023). However, as those were static images, it is conceivable that had these microtubules been observed for longer, they would have displaced the membrane and continued to elongate. For the centriculum to act as a microtubule filter, it must serve as a barrier for a subset of microtubules and impede their elongation. According to this model, an elongating microtubule encountering the centriculum would not penetrate the membrane, but exert force on it. If the centriculum has a given capacity to resist microtubule elongation, then making microtubules more stable or more abundant could result in a larger centriculum. Conversely, making microtubules weaker or less abundant could result in a smaller centriculum.

To test these possibilities, we used the *C. elegans* 1-cell embryo, which forms following fertilization. Centricula become visible as soon as the centrosome separate, and both centricula and centrosome increase in size throughout the cell cycle (Maheshwari et al., 2023). Because of that, the consequences of the various treatments described in this study were determined at metaphase, unless noted otherwise. At this stage, the parental pronuclei that encase the two parental genomes are adjacent to each other and the membrane interface between them is still visible (Fig 1A). Microtubules are nucleated from the outer region of the PCM and extend outwards (O’Toole et al, 2012), creating an accumulation of peri-centrosomal microtubules that appear as a “microtubule ring” (Fig 1A–C). To examine the consequences to the centriculum of stabilizing microtubules, we previously downregulated KLP-7, a microtubule depolymerase whose downregulation leads to enhanced microtubule outgrowth from centrosomes (Srayko et al, 2005). Indeed, downregulation of KLP-7 by RNAi resulted in elevated levels of peri-centrosomal microtubules (Maheshwari et al., 2023). Consistent with microtubules exerting force on the centriculum, and as shown previously (Maheshwari et al., 2023), downregulation of KLP-7 also led to an increase in the size of the centriculum, as determined by centriculum diameter and the area within the confines of the inner edge of the centriculum, referred to as centriculum void area (Fig 1B–E). Interestingly, while the diameter of the peri-centrosomal microtubule ring was significantly greater when KLP-7 was downregulated ((Maheshwari et al., 2023) and Fig 1F and G), the width of the microtubule ring did not increase, as measured but two orthogonal methods (Fig 1F): first, using thresholding, we determine the areas encased by the outer and inner edges of the tubulin ring using fluorescence intensity thresholding. Assuming these areas are perfect circles, we then calculated the radius of each. The difference between the two radii was defined as the width of the microtubule ring. Second, we created line scans across each tubulin ring and measured the width of the peaks at 90% maximal intensity. Microtubule ring width was defined as the average of the widths of the 4 peaks (two from each line scan). The thresholding (Fig 1H) and line scan (Fig 1I) methods revealed that the width of the microtubule ring did not increase, despite the increase in tubulin ring diameter. We previously showed that when the centriculum increases in size following *klp-7* RNAi, so does the underlying PCM (Maheshwari et al., 2023). Thus, it appears that in the case of KLP-7 downregulation, the cortical area of the PCM that is responsible for microtubule nucleation moves outwards as the PCM expands.

**Figure 2:**
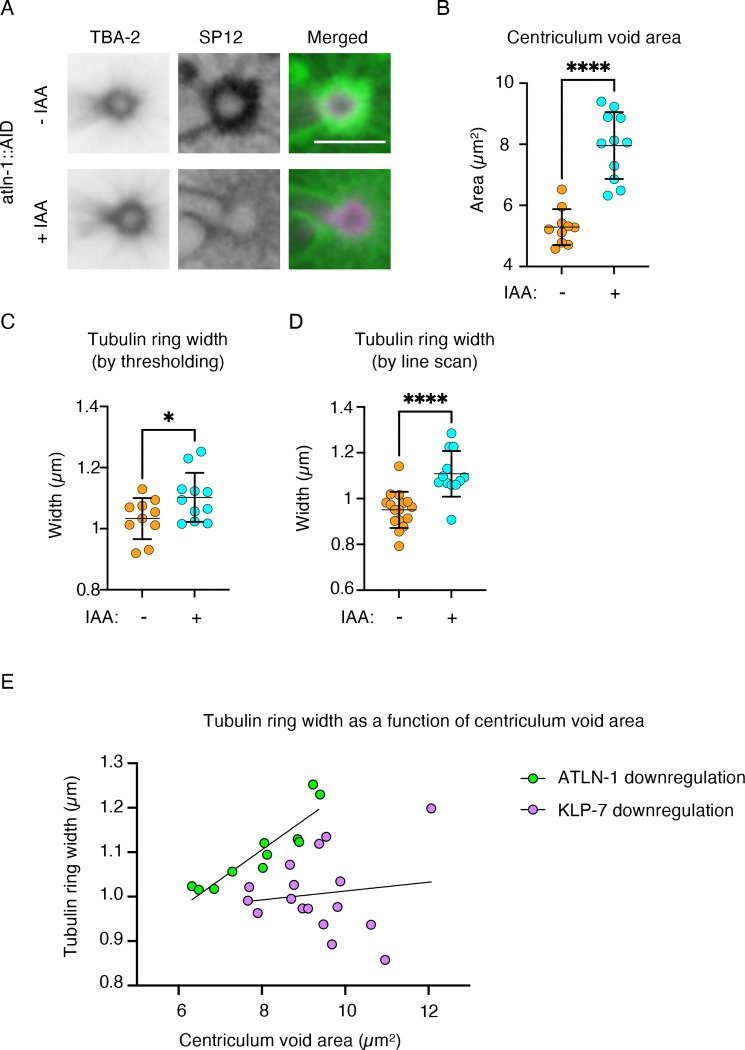
Downregulating ATLN-1 increases centriculum size and the width of the peri-centrosomal tubulin ring. **A)** Representative images of centricula from 1-cell embryos (OCF183) at metaphase expressing mCherry::SP12 (green in the merged images) and GFP::TBA-2 (magenta in the merged images), as well as ATLN-1::AID and the TIR1 ubiquitin ligase. Embryos were from worms were either left uncreated (top row) or treated with IAA to partially downregulate ATLN-1. Scale bar= 5 μm. **B)** Quantification of centriculum void, as described in Figure 1, of centricula from 1-cell embryos as shown in panel A, with (cyan; n=11) or without (orange; n=10) IAA treatment. p<0.0001 using the unpaired t test. Error bars indicate mean and standard deviation. **C and D)** Quantification of tubulin ring width using thresholding (C) or the line scan (D) methods in embryos from worms untreated (orange) or treated (cyan) with IAA. In panel C, n=10 and n=11 for − IAA and + IAA, respectively, p = 0.0462 using unpaired t test . In panel D, n=16 and n=12 − IAA and + IAA, respectively, p<0.0001 using the unpaired t test. Error bars indicate mean and standard deviation. **E)** Tubulin ring width following KLP-7 and ATLN-1 downregulation (purple and green, respectively) as a function of centriculum void area. Data plotted are from Fig 1E and H and Fig 2B and C. Tubulin ring widths depicted were determined by the thresholding method. Linear regression lines are shown for each data set. R^2^ for KLP-7 downregulation=0.01739; slope not significantly different from 0. R^2^ for ATLN-1 downregulation=0.8221, slope significantly different from 0 (p=0.0001).

Another condition that led to a larger centriculum is the partial downregulation of *C. elegans* atlastin, ATLN-1 ((Maheshwari et al., 2023) and Fig 2A–B), a crosslinker of ER tubules, which may contribute to centriculum integrity by increasing the density of the membrane lattice. Partial downregulation of ATLN-1 was done using an *atln-1:AID* degron construct, with or without a brief treatment with indole-3-acetic acid (IAA) ((Maheshwari et al., 2023); note that drastic downregulation of ATLN-1 results in embryos that cannot develop to metaphase). Our earlier measurements showed that centriculum diameter was 3.72 ± 0.17 μm (n=16) and 4.68 ± 0.46 μm (n=14) in the absence and presence of IAA, respectively (p<0.0001; (Maheshwari et al., 2023). The centriculum void was also greater when ATLN-1 is downregulated compared to control (Fig 2B). As in the case of *klp-7* RNAi, partial downregulation of ATLN-1 led to a tubulin ring with a larger diameter (2.05 ± 0.10 μm (n=16) and 2.43 ± 0.19 (n=14) in the absence and presence of IAA, respectively (p<0.0001; (Maheshwari et al., 2023)). However, unlike *klp-7* RNAi, the width of the tubulin ring was larger when ATLN-1 was partially downregulated compared to control (Fig 2C and D). Moreover, when ATLN-1 was partially downregulated, there was a good correlation between centriculum void area (which is a measure for the degree of ATLN-1 downregulation) and tubulin ring width (Fig 2E; R^2^=0.8221). There was no such correlation when KLP-71 was downregulated (Fig 2E; R^2^=0.0174, slope not statistically different from 0). There are at least two possible explanations for why downregulation of ATLN-1 leads to the expansion of the tubulin ring: first, it is possible that the PCM outer region from which microtubules are nucleated is expanded when ATLN-1 was partially downregulated. We think this possibility is less likely because ATLN-1 is not known to affect microtubules or centrosome proteins. An alternative explanation is that if partial downregulation of ATLN-1 results in centricula with fewer membrane junctions, microtubules might be able to penetrate deeper into the centriculum, resulting in longer peri-centromeric microtubules and a wider tubulin ring (see Discussion).

### Decrease in microtubule elongation or nucleation results in smaller centricula

**Figure 3:**
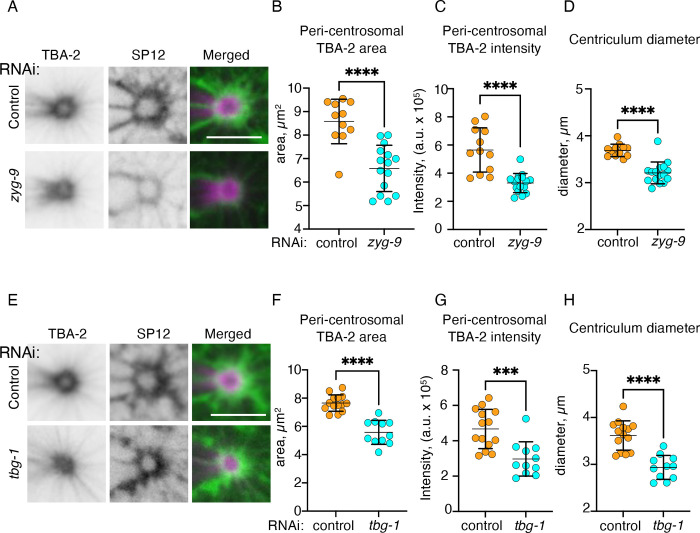
decrease in microtubule abundance leads to smaller centricula. **A)** Representative images of centricula from 1-cell embryos (OCF181) at metaphase expressing mCherry::SP12 (green in merged images) and GFP::TBA-2 (magenta in merged images), from worms treated with RNAi against a control (top row) or *zyg-9* (bottom row). Scale bar= 5 μm. **B-D)** Quantifications of GFP::TBA-2 peri-centrosomal area (panel B), GFP::TBA-2 total intensity (panel C) and average centriculum diameter (panel D) from embryos treated as shown in panel A. Panel B: n=11 for control (orange) and n=15 for *zyg-9* (cyan) RNAi treatments. p< 0.0001 using the Mann Whitney test. Panels C and D: n=12 for control (orange) and n=16 for *zyg-9* (cyan) RNAi treatments. p<0.0001 using unpaired t-test (panels C) or Mann Whitney test (panel D). Error bars represent mean and standard deviation. **E)** Representative images of centricula from 1-cell embryos (OCF181) at metaphase expressing mCherry::SP12 (green in merged images) and GFP::TBA-2 (magenta in merged images), from worms treated with RNAi against a control (top row) or *zyg-9* (bottom row). Scale bar= 5 μm. **F-H)** Quantification of peri-centrosomal GFP::TBA-2 area, total intensity and average centriculum diameter from the embryos treated as shown in panel E. n=14 for control (orange) and n=11 for *tbg-1* (cyan) RNAi treatment. p<0.0001 (panel F), p=0.0006 (panel I) and p<0.0001 (panel J) using unpaired t test. Error bars represent mean and standard deviation.

We next examined the consequences to centriculum size of either reducing microtubule elongation or nucleation. For the former, we downregulated ZYG-9, a microtubule polymerase and a homologue of *Xenopus* XMAP215 and human ch-TOG, which localizes to centrosomes and the mitotic spindle (Gard & Kirschner, 1987; Matthews et al, 1998). In *Xenopus*, XMAP215 has been shown to bind free tubulin dimers and catalyze their addition to the growing microtubule plus-end (Brouhard et al, 2008). Thus, cells in which ZYG-9 is downregulation are expected to form centrosome-nucleated shorter microtubules. Indeed, compared to control RNAi treatment, downregulation of ZYG-9 reduced the area occupied by peri-centrosomal microtubules, as observed by α-tubulin fused to GFP (GFP::TBA-2; Fig 3A and B). It also reduced the total intensity of GFP::TBA-2 in this area (Fig 3A and C). ZYG-9 depleted embryos also displayed significantly smaller centricula (Fig 3D), consistent with centriculum size being proportional to the microtubule force acting upon it.

Finally, we examined the effect on centriculum size of downregulating TBG-1, which codes for γ-tubulin, a conserved centrosomal component involved in microtubule nucleation (Moritz et al., 1995; Strome et al, 2001; Zheng et al., 1995). Although *C. elegans* centrosomes lacking TBG-1 can support some centrosome-dependent microtubule assembly (O’Toole et al, 2012), TBG-1 is the major microtubule nucleating factor at the centrosome, and downregulation of TBG-1 results in reduced microtubule nucleation rates, and hence fewer microtubules (Hannak et al, 2002). Similar to the effect of ZYG-9 downregulation, and consistent with the function of TBG-1, we found that both the area occupied by the peri-centrosomal microtubules, and total GFP::TBA-2 intensity within this area were reduced significantly after TBG-1 downregulation (Fig 3E–G). Moreover, reducing the levels of TBG-1 led to a significant reduction in centriculum diameter (Fig 3H). Taken together, these results show that the size of the centriculum is affected by microtubule stability and abundance, suggesting that when microtubules encounter the centriculum they exert force on it. Given that the majority of microtubules do not extend past the centriculum but remain adjacent to it in the form of pericentrosomal microtubules, a plausible explanation is that the centriculum impedes the elongation of microtubules that collide with it.

### Changes in centriculum size are accompanied by changes in PCM size

The above-mentioned results suggest that centriculum size could be directly regulated by microtubules. However, we previously observed that an increase in centriculum size due to downregulation of atlastin or KLP-7 is accompanied by an increase in PCM size, as determined by the area occupied by the PCM protein, SPD-5, the outermost component of the *C. elegans* PCM (Magescas et al., 2019; Maheshwari et al., 2023); Fig EV1A–C). To explore the generality of the observation that the PCM expands along with the centriculum, we examined whether the area of another PCM protein, TBG-1, also increases under conditions that increase centriculum size. As observed for GFP::SPD-5, downregulation of KLP-7 led to an increase in the area occupied by TBG-1::RFP (Fig EV1D–F). Interestingly, the amount of SPD-5, but not TBG-1, increases when the centriculum expands (Fig EV1G and H), as observed previously with ATLN-1 partial downregulation (Maheshwari et al., 2023). This suggests that centriculum size limits PCM size, as well as the amount of some proteins that are incorporated into the PCM.

**Figure 4:**
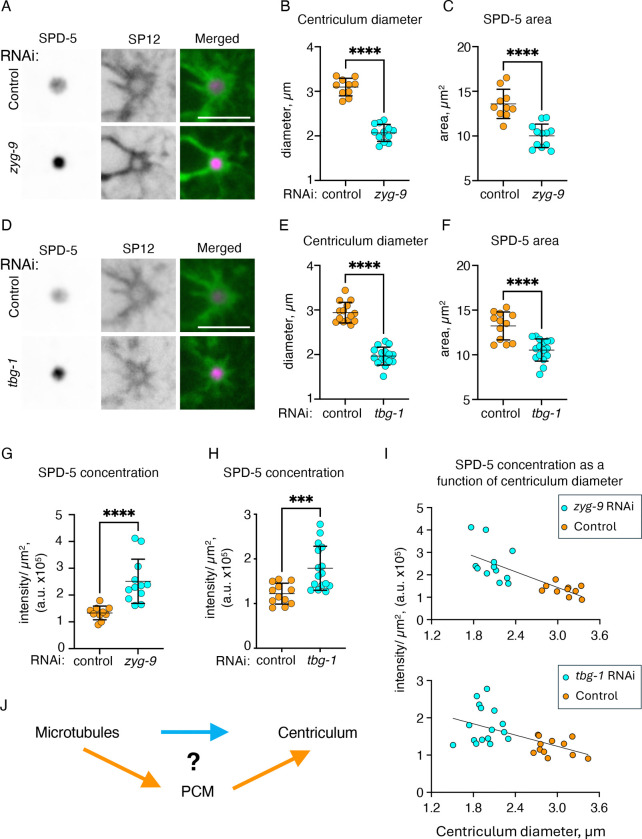
Manipulations that affect centriculum size also affect PCM size **A)** Representative images of centricula and PCM from 1-cell embryos (OCF176) at metaphase expressing mCherry::SP12 (green in merged images) and GFP::SPD-5 (magenta in merged images), from worms treated with RNAi against a control (top row) or *zyg-9* (bottom row). Scale bar= 5 μm. **B and C)** Quantification of average centriculum diameter and GFP::SPD-5 area from embryos treated as shown in panel A. n=10 for control (orange) and n=12 for *zyg-9* (cyan) RNAi treatments. p<0.0001 (panels B and C) using unpaired t test. Error bars indicate mean and standard deviation. **D)** Representative images of centricula and PCM from 1-cell embryos (OCF176) at metaphase expressing mCherry::SP12 (green in merged images) and GFP::SPD-5 (magenta in merged images), from worms treated with RNAi against a control (top row) or *tbg-1* (bottom row). Scale bar= 5 μm. **E-F)** Quantification of average centriculum diameter and PCM area from embryos treated as shown in panel D. Panel E: n=14 for control (orange) and n=16 for *tbg-1* (cyan) RNAi treatments. Panel F: n=12 for control (orange) and n=16 for *tbg-1* (cyan) RNAi treatments. p<0.0001 (panels E and F) using unpaired t test. Error bars indicate mean and standard deviation. **G and H)** Quantification of GFP::SPD-5 concentration, as determined by its total intensity divided by GFP::SPD-5 area (panels C and F) in control (orange), *zyg-9* (cyan, panel G), or *tbg-1* (cyan, panel H) RNAi treatments. p=<0.0001 (panel G) and p=0.0008 (panel H) using Mann Whitney test. Error bars indicate mean and standard deviation. **I)** GFP::SPD-5 concentration (panels G and H) as a function of centriculum diameter (panels B and E) in embryos following control (orange), *zyg-9* (cyan, top graph), or *tbg-1* (cyan, bottom) RNAi treatments. Linear regressions were performed through all the datapoints within a given graph. R^2^= 0.5644 and 0.3026 for the *zyg-9* and *tbg-1* experiments, respectively, and both slopes were significantly different from 0 (p<0.0001 and 0.0024 for the *zyg-9* and *tbg-1* experiments, respectively). **J)** Diagram depicting possible relationships between microtubules, the centriculum, and the PCM. See text for more detail.

We next examined whether the change in PCM size, as determined by GFP::SPD-5, extends to conditions that reduced centriculum size, namely ZYG-9 or TBG-1 downregulation. Indeed, in both cases, the decrease in centriculum size was accompanied by a decrease in PCM size (Fig 4A–F). Interestingly, while the area occupied by SPD-5 decreased, its concentration in the PCM, as determined by fluorescence intensity per μm^2^, increased (Fig 4G and H). This increase was inversely proportional to the diameter of the centriculum (Fig 4I; R2=0.5644 and 0.3026 for the *zyg-9* and *tbg-1* RNAi experiments, respectively). This suggests that a reduction in microtubule forces acting on the centriculum leads to compression of the PCM (see Discussion).

Taken together, our data are consistent with a model whereby microtubules affect centriculum size. This, in turn, affects PCM size, leading to PCM expansion when the centriculum is larger or compression when the centriculum is smaller. However, it was also possible that the effect of microtubules on the centriculum is indirect, occurring via the PCM. While PCM maturation is thought to be microtubule-independent (Hannak et al., 2002; Khodjakov & Rieder, 1999), the centrosome is subjected to pulling forces exerted by microtubules, although an effect on the PCM morphology is normally observed only at mitotic exit (Enos et al, 2018; Magescas et al., 2019; Mittasch et al, 2020), which is beyond the stage examined here. Nonetheless, it was formally possible that rather than the microtubules affecting centriculum size directly (Fig 4J, blue arrow), microtubules affected PCM size, and the PCM affected the size of the centriculum (Fig 4J, orange arrows). If the latter were the case, then a key condition of our microtubule filter hypothesis would not have been met. Thus, we needed to distinguish between the PCM vs. microtubules as the main contributor to centriculum size.

### The SPD-5 expansion mutant, in the presence of wild type SPD-5, reduces overall PCM size without affecting peri-centrosomal microtubules

**Figure 5:**
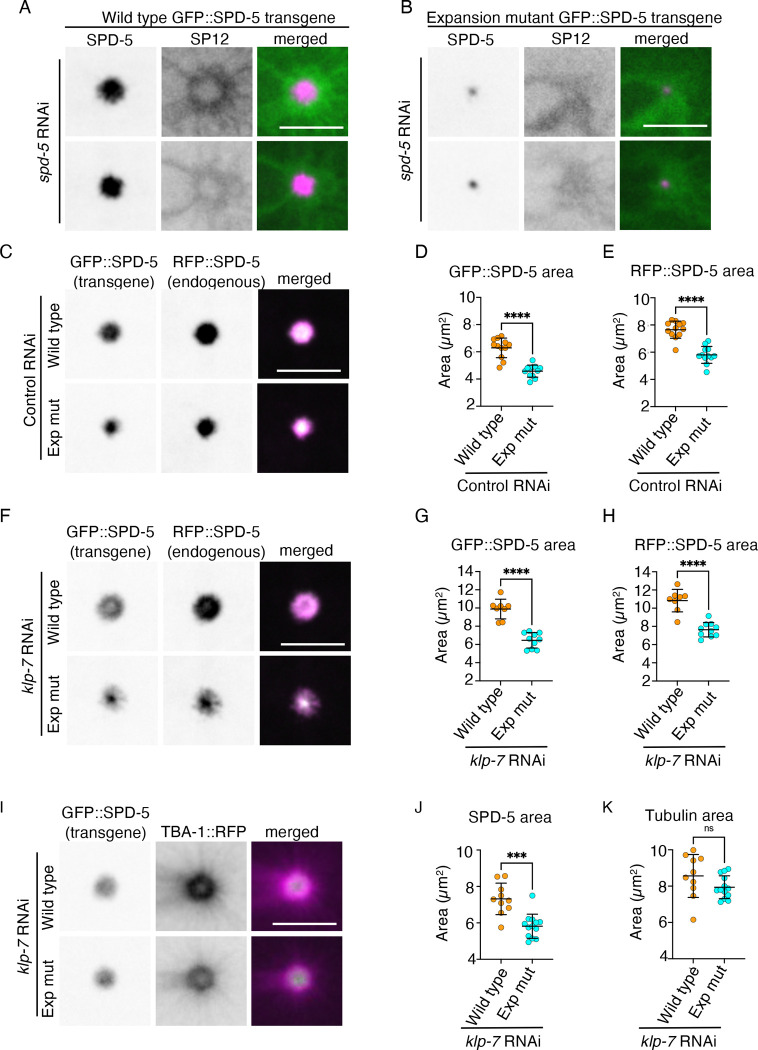
SPD-5 expansion mutant has dominant negative effect on PCM expansion. **A and B**) Representative images of centricula and PCM from two 1-cell embryos at metaphase expressing mCherry::SP12 (green in merged images) and either RNAi resistant GFP::SPD-5 wild type (panel A, magenta in merged image; OCF187) or RNAi resistant transgene GFP::SPD-5^exp^ (panel B, magenta in merged images; OCF189), after *spd-5* RNAi treatment. Scale bar= 5 μm. **C and F**) Representative images of centricula and PCM from 1-cell embryos at metaphase expressing endogenous RFP::SPD-5, and either transgenic GFP::SPD-5 (OCF201) or transgenic GFP::SPD-5^exp^ (OCF200), following control or *klp-7* RNAi treatment. Scale bar= 5 μm. **D, E, G, and H)** Quantification of endogenous RFP::SPD-5 and transgenic GFP::SPD-5 or SPD-5^exp^ areas after control (panels D and E) or *klp*-7 RNAi treatment (panel G and H), using the same conditions as in panels C and F. n= 12 for control RNAi. n=8 and 10 for wild type and expansion mutant for *klp-7* RNAi, respectively. p<0.0001 for all four graphs, using the Mann Whitney test for panel D and unpaired t test for panels E, G and H. Error bars indicate mean and standard deviation. **I)** Representative images of peri-centrosomal microtubules and PCM from 1-cell embryos at metaphase expressing endogenous TBA-1::RFP and either transgenic GFP::SPD-5 (OCF212) or transgenic GFP::SPD-5^exp^ (OCF213) after *klp-7* RNAi treatments. Images for the control RNAi condition are shown in Fig EV2A. Scale bar = 5 μm. **J and K)** Quantification of SPD-5 area and TBA-1 area from the embryos treated as shown in panel I. n=10 and n=13 for the transgenic wild type and expansion mutant SPD-5, respectively. p= 0.0006 (panel J) using the Mann Whitney test and p= 0.1188 (panel K) using the unpaired t test. Error bars indicate mean and standard deviation.

The *C. elegans* PCM component SPD-5 has two separable functions: (1) centrosome maturation and (2) microtubule nucleation via γ-tubulin recruitment (Ohta et al., 2021). Both functions are regulated by the PLK-1 kinase, which phosphorylates different residues on SPD-5. Ohta et al created an RNAi-resistant *spd-5* phospho-mutant allele, *spd-5*^*exp*^, carrying S653A and S658A substitutions, which they expressed as a transgene in the presence of the endogenous wild type, RNAi sensitive, SPD-5. When SPD-5^exp^ was expressed as the sole SPD-5 version, the expansion of the PCM was greatly attenuated compared to a strain expressing transgenic wild type SPD-5 as its sole source of SPD-5 (Ohta et al., 2021) and Fig 5A and B), suggesting that phosphorylation of S653 and S658 is required for PCM expansion (Ohta et al., 2021). In the presence of the endogenous wild type SPD-5, transgenic SPD-5^exp^ occupied a greater area than without the endogenous protein (compare transgenic GFP::SPD-5^exp^ in Fig 5B, without endogenous SPD-5, to Fig 5C, with endogenous SPD-5). This suggests that SPD-5^exp^ is able to integrate into the wild type SPD-5 lattice, despite lacking two PLK-1 phosphorylation sites that contribute to PCM expansion. This is consistent with findings by Wueseke et al, who made similar observation with a *spd-5* allele lacking four PLK-1 phosphorylation sites, including the two in SPD-5^exp^ (Wueseke et al., 2016). As in Wueseke et al, we also observed a good overlap between the endogenous SPD-5 (fused to RFP) and transgenic SPD-5 or SPD-5^exp^ proteins (both fused to GFP) (Fig 5C and F), suggesting that both transgenes are integrated throughout the SPD-5 lattice. Interestingly, the expression of the SPD-5^exp^ in the presence of the endogenous, wild type RFP::SPD-5, led to a smaller overall PCM (Fig 5C–E). The average PCM size in the presence of the SPD-5^exp^ transgene, as determined by the endogenous SPD-5, was 75% that of the PCM size in the presence of the wild type SPD-5 transgene. This defect was slightly enhanced when microtubules were stabilized by KLP-7 downregulation, where the PCM was larger, but the average PCM size in the presence of the SPD-5^exp^ transgene was 69% of the control (Fig 5F–H). A similar effect on PCM size by the SPD-5 expansion mutant was observed by Wueseke et al (Wueseke et al., 2016). Thus, SPD-5^exp^ may act as a dominant negative, impeding the ability of the SPD-5 lattice to fully expand. Alternatively, or in addition, the presence of SPD-5^exp^ could have led to reduced levels of wild type SPD-5, as proposed by Wueseke et al (Wueseke et al., 2016), resulting in a smaller PCM.

The presence of endogenous SPD-5 under conditions where the PCM was smaller (namely with SPD-5^exp^ expression) raised the possibility that microtubule nucleation would not be drastically affected by the presence of the SPD-5^exp^ mutant. To examine this, we expressed TBA-1::RFP in the presence of endogenous SPD-5, and either the wild type or SPD-5^exp^ transgene fused to GFP. As before, the PCM was smaller in the presence of the SPD-5^exp^ mutant, either when KLP-7 was downregulated (Fig 5I and J) or under control RNAi conditions (Fig EV2A and B). However, the area occupied by the peri-centrosomal microtubules (namely the outer edge of the tubulin ring) was indistinguishable when either transgenic wild type or SPD-5^exp^ were present (Fig 5K for KLP-7 downregulation and Fig EV2C for control RNAi). Moreover, in the presence of the SPD-5^exp^ mutant, microtubules extended past the PCM under control RNAi conditions (Fig EV2B and C), and especially when KLP-7 was downregulated (Fig 5J and K; note the difference in area between the pericentrosomal microtubules/TBA-1 and the PCM/SPD-5 in the presence of SPD-5^exp^). Taken together, the presence of SPD-5^exp^ created a situation where the PCM was smaller, but the size of the tubulin ring was the same as the control. This allowed us to examine whether the centriculum “rests” on the PCM or on microtubules, as discussed next.

### The inner edge of the centriculum is adjacent to peri-centrosomal microtubules, not the PCM

**Figure 6:**
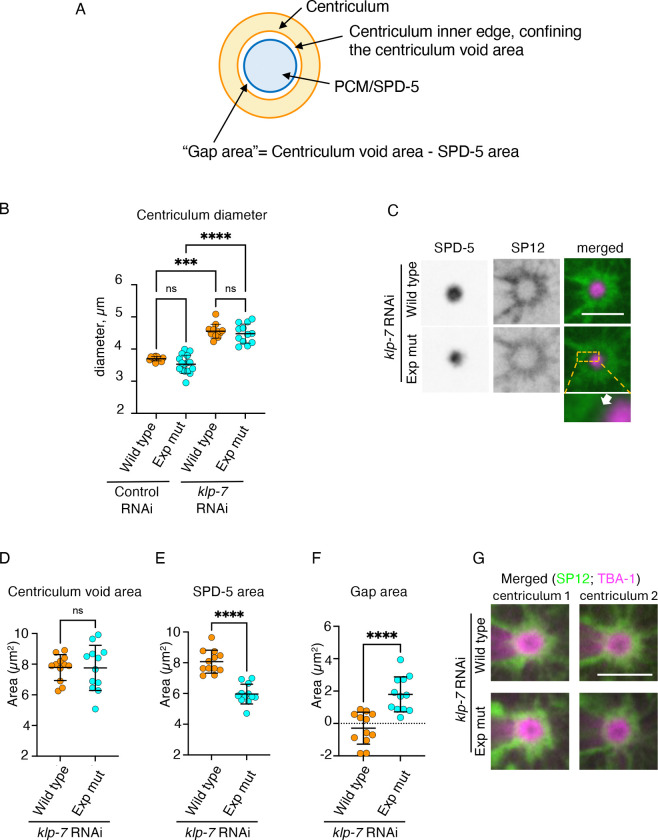
Centriculum size correlates with the position of peri-centrosomal microtubules, not the PCM. **A)** Diagram showing how the “gap area” was derived. **B)** Quantification of centriculum diameter from 1-cell metaphase embryos expressing mCherry::SP12 and either transgenic wild type GFP::SPD-5 (strain OCF187, “wild type”) or transgenic GFP::SPD-5^exp^ (strain OCF189, “exp mut”) following control or *klp-7* RNAi treatment. n=12, for wild type under control RNAi, n=16 for exp mut under control RNAi, n=12 for wild type under *klp-7* RNAi and n=12 for exp mut under *klp-7* RNAi. p values, as determined by Kruskal-Wallis test were as follows: >0.9999 for wild type vs exp mutant, control RNAi, >0.9999 for wild type vs exp mutant, *klp-7* RNAi, 0.0009 for wild type, control vs *klp-7* RNAi, <0.0001 for exp mut, control vs *klp*-7 RNAi. Error bars indicate mean and standard deviation. **C)** Representative images of centricula and PCM from 1-cell embryos at metaphase expressing mCherry::SP12 (green in merged images) and either transgenic wild type GFP::SPD-5 (magenta in merged images; strain OCF187) or transgenic GFP::SPD-5^exp^ (magenta in merged images; strain OCF189) following *klp-7* RNAi treatment. White arrow in the bottom merged image points to a gap between the centriculum and the PCM/SPD-5. Additional images, as well as images from control RNAi conditions are shown in Fig EV2D and E. **D, E, and F**) Quantification of centriculum void area, SPD-5 area, and the gap area from embryos treated as shown in panel C. p values were 0.9620 (panel D), <0.0001 (panel E) and <0.0001 (panel F), using unpaired t test. n=12 and 12 for transgenic wild type (orange) and SPD-5 exp mutant (cyan), respectively. Quantification of the same parameters under control RNAi conditions is shown in Fig EV2F–H. **G)** Examples of centricula and peri-centrosomal microtubules from 1-cell embryos expressing SP12::GFP (green in merged image) and TBA-1::RFP (magenta in merged image) and either transgenic wild type SPD-5 (OCF214) or SPD-5^exp^ (OCF215), following *klp-7* RNAi treatment. Images from control conditions and additional images following *klp*-7 RNAi are shown in Fig EV2I and J. Scale bar = 5 μm.

To determine whether centriculum size correlates with the area occupied by the PCM or peri-centrosomal microtubules, we first used a strain expressing the fluorescently-tagged centriculum marker mCherry::SP12, also expressing either transgenic SPD-5 or SPD-5^exp^ fused to GFP, in the presence of endogenous SPD-5. Using mCherry::SP12, we measured two centriculum parameters: centriculum diameter and centriculum “void area” (Fig 1B and 6A). We then defined the “gap area” as the difference between the centriculum void area and the area occupied by SPD-5, representing the PCM. If the centriculum “rests” on the PCM, the gap area should be close to zero in the presence of with either transgenic SPD-5 or SPD-5^exp^, and the centriculum would be smaller in the SPD-5^exp^ strain, which has a smaller PCM (Fig 5C–J and EV2B). If, however, the size of the centriculum is determined by microtubules, then in the presence of transgenic SPD-5^exp^, where the microtubules extend beyond the PCM (Fig 5J and K), the gap area should be greater than zero. Moreover, if the centriculum rests on microtubules, then centriculum size should the same in the presence of transgenic SPD-5 or SPD-5^exp^, since the area occupied by peri-centrosomal microtubules is the same in both strains (Fig 5K and EV2C). Our data are consistent with the latter possibility, where the size of the PCM is determined by microtubules: first, the diameter of the centriculum was the same regardless of which transgene was expressed, mirroring the behavior of pericentrosomal microtubules rather than the PCM (Fig 6B). As expected, centriculum diameter was greater in the presence of RNAi against *klp-7*, again with no difference between the transgenes (Fig 6B). Second, a gap was clearly visible between the PCM and the centriculum, especially when KLP-7 was downregulated (Fig 6C and Fig EV2D and E). The calculated size of the gap, namely the difference between the centriculum void and PCM areas, was significantly greater in the presence of *spd-5*^*exp*^ (Fig 6D–F and Fig EV2F–H; note that the negative values for the gap area stem from the overlap in fluorescence between the tubulin ring and the centriculum). We next examined the location of peri-centrosomal microtubules relative to the centriculum, using embryos from worms expressing SP12::GFP and TBA-1::RFP, along with transgenic wild type SPD-5 or SPD-5^exp^. Unlike the situation with PCM, there was no gap between the pericentrosomal microtubules and the centriculum, using either control RNAi or *klp-7* RNAi (Fig 6G and Fig EV2I and J). Taken together, our data are consistent with a model whereby the centriculum abuts the peri-centrosomal microtubules, not the PCM.

### Centriculum porosity correlates with the ability of microtubules to traverse the centriculum.

At metaphase, two types of microtubules extend beyond the peri-centrosomal microtubule ring: astral microtubules, which extend towards the cell cortex, and spindle microtubules, which extend towards the chromosomes. The density of spindle microtubules is much greater than that of astral microtubules (Fig 1A and see below). If the centriculum serves as a microtubule filter, we would expect that it would be more permissive to passage of microtubules on the side facing the chromosomes. We therefore set out to characterize the structure of the centriculum facing the cortex vs. facing the chromosomes.

**Figure 7:**
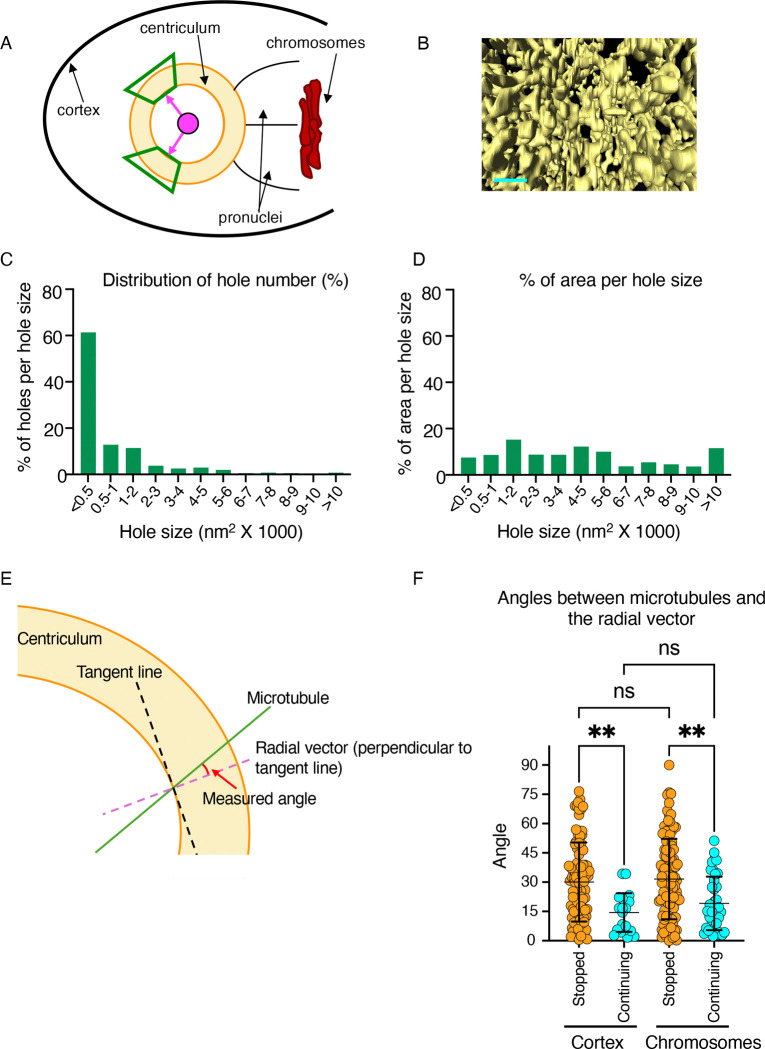
Porousness of the centriculum facing the cortex. **A)** Diagram depicting the centriculum (in yellow) fused to the two pronuclei (black lines) at a metaphase 1-cell embryo. Chromosomes and the cortex are also indicated. The areas analyzed for pore size are shown as green trapezoids. A central fiducial (in pink) that is equidistance (pink arrows) from the areas analyzed is also shown. **B)** A representative image of a segmented area based on FIB-SEM data (Maheshwari et al., 2023) on the cortical side of the centriculum, taken from the fiducial mark as depicted in panel A. Additional examples are shown in Fig EV3B. Scale bar= 200 nm. **C)** Binned frequency distribution of holes present on the cortical side of metaphase centricula in a 1-cell embryo. Bin size range is shown on the X axis. n= 507 from 6 images taken from 3 centricula. **D)** Binned frequency distribution of the percentage of total open area per hole size range, using the same data as in panel C. **E)** Diagram depicting the angle of microtubules (green line) passing through the centriculum (in yellow) relative to the radial vector (dashed purple line). The actual measurement was done between the microtubule and the tangent line (dashed black line) and the value obtained was subtracted from 90 to obtain the angle to the radial vector, which is perpendicular to the tangent line. **F)** Quantification of angles between of stopped (in orange) and continuing (in cyan) microtubule and radial vector for astral (cortex-facing) and spindle (chromosome-facing) microtubules. Based on EM tomography data (Redemann et al., 2017). p= 0.0029 (for cortex-facing) and 0.0018 (chromosome-facing) using One-way Anova with multiple comparisons. Error bars indicate mean and standard deviation.

To this end, we measured the effective pore size that is available for microtubules nucleated at centrosomes to pass through the centriculum, using volume electron microscopy data acquired via focused ion beam-scanning electron microcopy (FIB-SEM; (Maheshwari et al., 2023)). Starting with the centriculum side facing the cortex, we created a fiducial in the center of segmented metaphase centricula, and imaged a portion of the centriculum against a black background (Fig 7A and B; images were taken from within the centrosome towards the cortex). To maximize having the openings imaged *en face*, the angles between the center line and the top or bottom of any image were kept to a minimum (23.8° ± 2.82°; average and standard deviation, n=12). We then determined the area of each opening, defined as “hole”, that can be seen through the centriculum. Considering that the diameter of a microtubule is ~25 nm, the area of the smallest hole through which a microtubule can pass would be 490 nm^2^ (*π* × 12.5^2^) and probably larger since most holes are not perfect circles. The fraction of cortex-facing centriculum area that was open, namely the sum of all hole area out of the total area imaged, was 7.85 ± 1.66% (n=6, from 3 centricula). The cross sections of 60% of these holes, accounting for 7.5% of the total open area, were below 500 nm^2^, likely too small to allow for a microtubule to pass (Fig 7C and D). However, a significant fraction of holes had a cross-section area that was large enough to accommodate one or more microtubules (Fig 7C), with roughly 10% of the open area being in holes that were larger than 10,000 nm^2^ (Fig 7D).

Given that only a small fraction of the centriculum area facing the cortex is available for the microtubules to go through, and the fact that the thickness of centriculum at metaphase is around 1 μm (Maheshwari et al., 2023), we predicted that the microtubules that become fully elongated astral microtubule are those that take the shortest path through the centriculum, and thus less likely to encounter centriculum membrane. To test this hypothesis, we used ER tomography datasets of 1-cell embryos at metaphase (Maheshwari et al., 2023; Redemann et al., 2017) and measured the angle of microtubules as they pass the inner boundary of the centriculum relative to the radial vector- the line that is perpendicular to the tangent line (Fig 7E). The shortest path through the centriculum would be that of the radial vector (angle= 0°); increasing angles between a microtubule path and the radial vector would mean longer paths through the centriculum. We measured the angles of 106 microtubules that ended within the centriculum (“stopped” microtubules), and 21 of the less prevalent microtubules that passed all the way through (“continuing” microtubules). Consistent with our model, the distribution of angles of microtubules that passed all the way through the centriculum was significantly narrower, and closer to the radial vector, than the range of angles for microtubules that ended within the centriculum (Fig 7F). Taken together, our data suggest that the centriculum serves as a passive microtubule filter, allowing only a subset of microtubules to extend pass the centriculum, into the cytoplasm.

**Figure 8:**
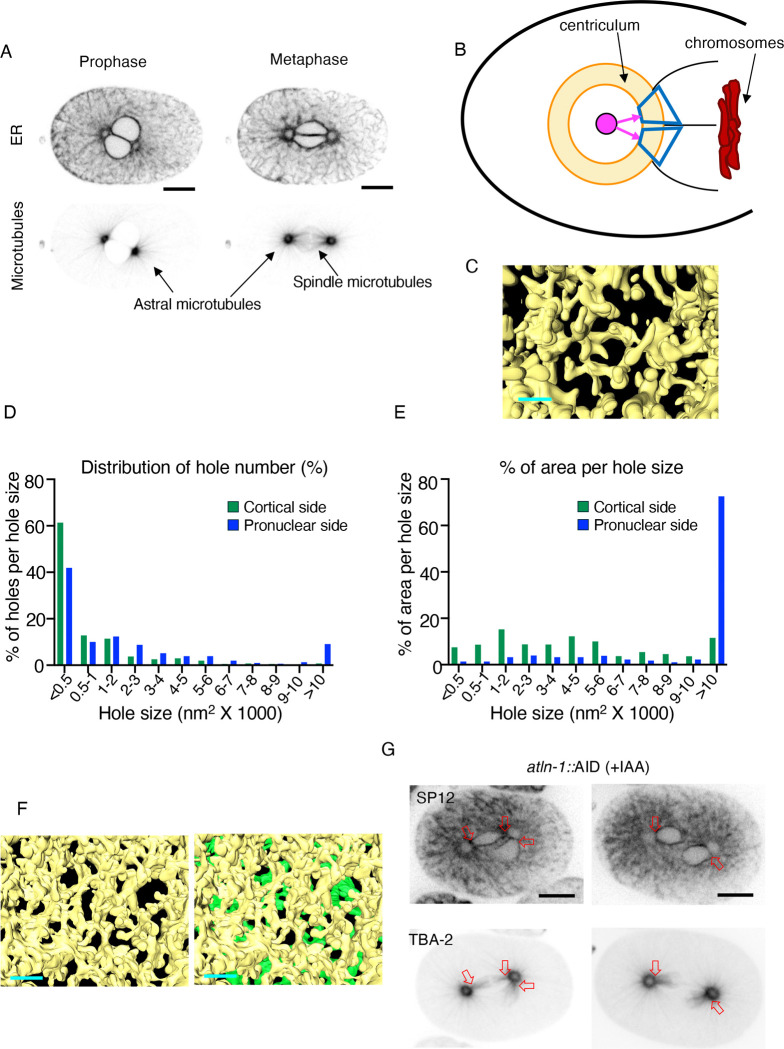
The porosity of the centriculum on the pronuclear side is greater than on the cortical side. **A)** Representative images of 1-cell *C. elegans* embryos expressing mCherry::SP12 to visualize the centriculum and GFP::TBA-2 to visualize microtubules (OCF181) at prophase (left side) and metaphase (right side). Note that at prophase, microtubules are excluded from the nucleoplasm, and that in metaphase, the density of spindle microtubules is greater than astral microtubules. **B)** Diagram depicting the centriculum (in yellow) fused to the two pronuclei (black lines) at a metaphase 1-cell embryo. The areas analyzed for pore size in this experiment are on the pronuclear side and shown as blue trapezoids. A central fiducial (in pink) that is equidistance (pink arrows) from the areas analyzed is also shown. **C)** A representative image of a segmented area based on FIB-SEM data (Maheshwari et al., 2023) of a pronuclear area of a metaphase centriculum. Additional images are shown in Fig EV3B, where the openings on the cortex and pronuclear sides and can be compared qualitatively. Scale bar = 200 nm. **D)** Binned frequency distribution of holes present on the cortical side (in green) and pronuclear side (in blue) of metaphase centricula in a 1-cell embryo. The data for holes on the cortical size are the same as shown in Fig 7C. Bin size range is shown on the X axis. n=308 for pronuclear side holes from 6 images taken from 3 centricula. **E)** Binned frequency distribution of the percentage of total open area per hole size range, for holes on the cortical side (green) and the pronuclear side (blue) using the same data as in panel D. The data for holes on the cortical size are the same as shown in Fig 7D. **F)** Left panel: A representative image of a segmented area based on FIB-SEM data (Maheshwari et al., 2023) from the pronuclear side of a prometaphase centriculum (in yellow), visualized from the center of the centrosome. Right panel: the same image with segmentation of nuclear membrane remnants (in green) that are between the centriculum and the prometaphase chromosomes (not shown). Additional images are shown in Fig EV3E. Scale bar= 200 nm. **G)** Two 1-cell embryos expressing mCherry::SP12 and GFP::TBA-2 from worms in which atlastin was downregulated using an auxin-inducible degron (AID) system (OCF183, see (Maheshwari et al., 2023). Red arrows point to sites were centricula fused with the pronuclei (top panels). The same arrows are shown in the bottom panels, where they are adjacent to sites were spindle microtubules extend towards the chromosomes. Note that unlike the wild type situation (Fig 8A), under these conditions the two pronuclei fail to align and form an extensive pronuclear interface membrane. Scale bar= 10 μm.

To form a spindle, microtubules must exit the centrosome and enter the space around the chromosomes. Prior to metaphase, an intact nuclear envelope prevents centrosome-nucleated microtubules from entering the nucleoplasm (Fig 8A, prophase). Just before metaphase, two events take place: the nuclear envelope loses its integrity specifically next to centrosomes (Hachet et al, 2012), and the centriculum fuses with the nuclear membrane (Maheshwari et al., 2023). As noted earlier, the density of spindle microtubules exceeds that of astral microtubules (Fig 8A, metaphase). If the centriculum acts as a microtubule filter, then its porosity on the spindle side, facing the chromosomes, should be greater than its porosity on the astral microtubule side, facing the cortex. To examine this, we repeated the analysis of centriculum holes described above, but on the side facing chromosomes (Fig 8B). As before, we kept the angle between the center line and the top or bottom of all images to a minimum (22.8° ± 2.23°; average and standard deviation, n=12). Qualitatively, it already appeared that the holes on the chromosomal side are larger than those on the cortical size (compare Fig 8C and Fig 7B, also Fig EV3A and B). This was corroborated by quantitative analyses showing that (a) The total open area of the centriculum facing the chromosome was 20.66 ± 3.75%, (n=6 from 3 centricula), compared to 7.85 ± 1.66% on the cortical side, (b) a smaller fraction of holes on the chromosomal side had a cross-section area of less than 500 nm^2^ (Fig 8D; roughly 40% on the chromosomal side compared to 60% on the cortical side), (c) a greater fraction of holes were larger than 10,000 nm^2^ (Fig 8D; roughly 9% on the chromosomal side compared to 0.8% on the cortical side), and (d) the majority of open space on the pronuclear side was in the form of holes greater than 10,000 nm^2^ (Fig 8E; 72.5% on the chromosomal side compared to 11.5% on the cortical side). When comparing the angles of stopped vs continuing microtubules relative to the radial vector, as was done for microtubules on the cortex side (Fig 7F), the angle distribution of continuing microtubules (n=40) on the chromosome side was again narrower than stopped microtubules (n=106), although the angle distributions themselves of stopped and continuing microtubules were the same on both sides of the centriculum (Fig 7F). Nonetheless, the porosity of the centriculum on the chromosomal side was much greater than on the cortical side, providing an explanation for how, at metaphase, spindle microtubules can achieve a greater density compared to astral microtubules.

Whether the different degrees of porosity are caused by centriculum geometry or by properties of spindle microtubules remains to be determined (see Discussion). In this regard, the larger hole size of the centriculum on the chromosomal side is unlikely to be the only reason that spindle microtubule density is greater than that of astral microtubules. At prometaphase, the percent of open centriculum area (14.64 ± 4.14%, n=6 from 3 centricula), percent of holes per hole size, and percent area per hole size (Fig EV3C and D) on the chromosomal side of the centriculum were similar to those of metaphase centricula. However, the effective hole available for microtubules to reach the chromosomes is significantly smaller because at prometaphase, remnants of the nuclear membrane are still present, creating an additional barrier for microtubules to enter the nucleoplasm (Fig 8F and Fig EV3E, note the presence of nuclear membrane (in green) masking many of the openings). The difference in the relationship between the centriculum and the nuclear membrane at prometaphase and metaphase is that at prometaphase the centriculum is still adjacent to the nuclear membrane, with a few possible contact sites (see, for example Fig EV3F), but no membrane junctions that indicate actual fusion. In contrast, at metaphase, the centriculum is completely fused with the remnants of the nuclear membrane (Maheshwari et al., 2023), possibly in an atlastin-dependent manner ((Araújo et al., 2023) and see below). Therefore, at metaphase, there are no other membranes between the centriculum and the chromosomes. Thus, the fusion of the centriculum and mitotic remnants of the nuclear membrane may reduce membrane barriers for spindle formation. A further indication that the centriculum-nuclear membrane fusion is important for spindle microtubule formation is the state of metaphase 1-cell embryos following atlastin downregulation. Under these conditions, for unknown reasons, pronuclei have difficulties aligning, and they sometimes fuse with only one of the two centricula (Fig 8G), leading to the formation of monopolar spindles. Importantly, under these conditions, dense microtubules can be observed only where the centriculum is fused to the pronuclei, while the rest of the centriculum is surrounded by lower density, astral microtubules. Taken together, we proposed that formation of high-density microtubules on the pronuclear side of the centriculum is facilitated by two processes: the presence of large openings that traverse the centriculum, and the fusion of the centriculum with remnants of the nuclear membrane.

## Discussion

**Figure 9:**
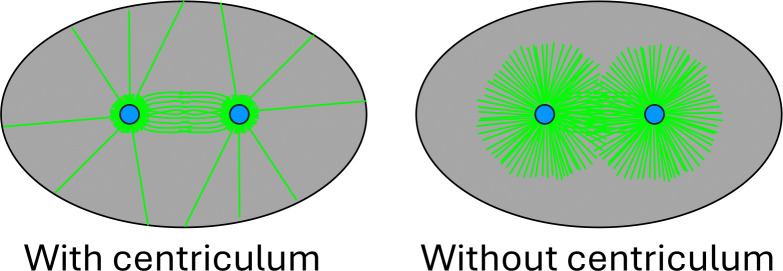
A model for the effect of the filter function of the centriculum on astral microtubules in the *C. elegans* 1-cell embryo at metaphase. Centrosomes are shown in blue and microtubules are shown in green. See text for detail.

The centriculum is a prominent, ER-derived dense membrane reticulum that surrounds centrosomes in *C. elegans* early embryos (Maheshwari et al., 2023). Similar ER-derived membrane accumulation has been observed in other organisms (Araújo et al., 2023; Bergman et al., 2015; Diaz et al., 2019; Harris, 1975; Karabasheva & Smyth, 2019; Waterman-Storer et al., 1993), but whether their ultrastructure is same as the *C. elegans* centriculum remains to be determined. We have previously shown that increasing centriculum size through atlastin partial downregulation, likely reducing ER tubule junction formation, led to an increase in centrosome size, tubulin diameter and microtubule nucleating capacity (Maheshwari et al., 2023). This observation provided evidence that in early embryogenesis, the *C. elegans* centrosome is affected by the membrane adjacent to it. Here we show that the size of the centriculum is affected by the number and stability of centrosome-nucleated microtubules (Fig 1–3), suggesting that microtubules exert force on the centriculum. This further suggests that upon impact, microtubules cannot simply move the centriculum membrane out of their path, although when ATLN-1 is downregulated, microtubules may be able to penetrate deeper into the centriculum (Fig 2), possibly due to the presence of fewer membrane tubule junctions. While elongating microtubules have the capacity to distort a membrane sheet (for example, see (Zimmerman & Chang, 2005)), we propose that the reticular nature of the centriculum allows it to resist forces exerted on it by microtubules. Consistent with this possibility, when ATLN-1 is partially downregulated, presumably leading to a less dense reticulum, the centriculum is larger than in wild type embryos, potentially due to forces exerted by microtubules on a weaker reticular structure. Consequently, the centriculum can act as a microtubule filter, blocking the majority of centrosome-nucleated microtubules to create an accumulation of short, peri-centrosomal microtubules, and allowing only a subset of microtubules to become fully extended astral or spindle microtubules. A similar accumulation of peri-centrosomal microtubules was observed in other organisms that also have peri-centrosomal ER accumulation (Kiyomitsu et al., 2024; Rollins & Blankenship, 2023; Xie et al., 2025), suggesting that the filter function of the centriculum may be a general phenomenon. Had we been able to eliminate the centriculum, we predict that more astral microtubules would have extended, potentially leading to competition on limited cytoplasmic pools of soluble tubulin and reducing the ability of astral microtubule to extend all the way to cortex (Fig 9).

In the process of examining the effect of reduced microtubule abundance on the centriculum, we observed that decreasing centriculum size, by reducing microtubule number or stability, led to a smaller PCM (Fig 4). Given that under these conditions the fluorescence intensity per area of SPD-5 increased (Fig 4G–I), we propose that the decrease in centriculum size led to compression of the PCM. This is an intriguing possibility, as it suggests that the PCM lattice is malleable, able to expand or fold inwards in response to forces acting upon it, akin to a Hoberman Sphere (https://www.hoberman.com/portfolio/hoberman-sphere-toy/). That the PCM is malleable was also proposed by Laos et al, who noted that the PCM lattice *in vivo* can grow by incorporating SPD-5 anywhere within the lattice (Laos et al., 2015). This property could contribute to the PCM’s ability to deform in response to forces exerted on it by astral microtubules (Enos et al., 2018), where the PCM could stretch rather than break. This property, known as ductility, was also proposed by Mittasch et al based on the PCM’s deformability during anaphase in response to external forces (Mittasch et al., 2020).

Normally, in the early *C. elegans* embryos, a significant fraction of microtubules does not extend far beyond the centrosome, creating, by fluorescence microscopy imaging, a ring of microtubules around the centrosome. This ring, referred to here as pericentrosomal microtubules, overlaps with outermost part of the PCM (Magescas et al., 2019). This organization prevented us from distinguishing between microtubules and PCM as the main factor contributing to centriculum size. To create a situation where PCM size is smaller without affecting the area occupied by peri-centrosomal microtubules, we expressed a *spd-5* allele, *spd-5*^*exp*^, that is defective in PCM expansion (Ohta et al., 2021), and observed that the resulting SPD-5 lattice was smaller than its size in the wild type situation (Fig 5). An inhibitory effect by SPD-5 mutant proteins on the SPD-5 lattice has been observed previously: Nakajo et al observed this phenotype with a 272 amino acid C-terminal deletion, which did not encompass the residues mutated in SPD-5^exp^ (Nakajo et al., 2022), while Wueseke et al observed it with a SPD-5 mutant lacking four PLK-1 phosphorylation sites, *spd-5*^*4A*^, including the two in SPD-5^exp^ (Wueseke et al., 2016). Rios et al found that PLK-1 phosphorylation of SPD-5 reduces intramolecular interactions, allowing these domains to interact intermolecularly (Rios et al., 2024). We therefore postulate that the SPD-5^exp^ mutant could be folded in such a way that interferes with its ability to form intermolecular connections and thus impede the expansion of the SPD-5 lattice. The presence of transgenic mutant *spd-5* may have led to reduced levels of endogenously expressed *spd-5* (Wueseke et al., 2016), thus resulting in a smaller PCM.

Regardless of how SPD-5^exp^ interfered with SPD-5 expansion, the smaller PCM in the presence of SPD-5^exp^ allowed us to ask whether the centriculum is adjacent to microtubules or the PCM. There was no appreciable difference in the area occupied by peri-centrosomal microtubules in the presence of transgenic wild type SPD-5 vs SPD-5^exp^ (Fig 5I–K), likely due to a sufficient amount of endogenous wild type SPD-5, and because SPD-5^exp^ retained some of its γ-tubulin recruitment ability (Ohta et al., 2021). Likewise, there was no difference in the size of the centriculum in the presence of transgene-expressed wild type vs. SPD-5^exp^ (Fig 6B). Under conditions where the PCM was smaller, a gap could be observed between the PCM and the centriculum, but not between the pericentrosomal microtubules and the centriculum (Fig 6 and Fig EV2). We interpret this observation to mean that normally, the centriculum “rests” on microtubules. Consistent with this interpretation, the centriculum collapses when microtubules are depolymerized at metaphase (Maheshwari et al., 2023).

We further postulate that centriculum-microtubule interactions impede microtubule elongation, resulting in the observed ring formed by the peri-centrosomal microtubules. It is formally possible that the abundance of short microtubules in the vicinity of the centrosome is independent of the presence of the centriculum, caused by an inherent property of these microtubules or due to microtubule-associated proteins. In this scenario, the centriculum simply “rests” on these microtubules, without affecting their length. We cannot examine this experimentally because to date, there are no conditions that eliminate the centriculum without affecting microtubules. However, three observations argue against this possibility: First, downregulation of atlastin, which forms 3-way ER tubule junctions, is not expected to affect microtubule properties. Under these conditions, however, the centriculum is enlarged, as is the width of the peri-centrosomal microtubule ring (Fig 2). While it is formally possible that the area from which microtubules are nucleated within the PCM has expanded, we favor the interpretation that the downregulation of atlastin change centriculum properties such that microtubules could elongate further into the centriculum. This would not be the expected result if the length of peri-centrosomal microtubules was an inherent property of these microtubules. Second, if microtubules were agnostic to the presence of the centriculum, there would be no difference in the angle relative to the radial vector of microtubules that terminate within the centriculum and those that extend past it. However, we find that the angle distribution of microtubules that extend past the centriculum is significantly narrower, and the average angle closer to 0, than microtubules that terminate within the centriculum (Fig 7E and F). Finally, numerous in vitro studies have examined microtubule length, either directly or indirectly, and in none of them are microtubules as short as the *C. elegans* 1-cell peri-centrosomal microtubules (Arnal et al, 2004; Belmont et al, 1990; Chrétien et al, 1995; Farrell et al, 1987; Fygenson et al, 1994; Gould & Borisy 1977; Grego et al, 2001; Kuriyama & Borisy, 1981; Mitchison & Kirschner, 1984; Verde et al, 1990). This suggests that the centriculum, due to its width, serves as a passive filter, where microtubules that travel a shorter distance are more likely to traverse the centriculum and become elongated astral microtubules. Taken together, we propose that the reason for the accumulation of short microtubules in the vicinity of the centrosome in the *C. elegans* embryo is the presence of the centriculum, which impedes microtubule elongation, eventually leading to microtubule catastrophe. This, in turn, would create an environment of high tubulin concentration, as observed by Baumgart et al (Baumgart et al., 2019), that is captured by the PCM, which is capable of concentrating tubulin in *in vitro* (Woodruff et al., 2017).

At metaphase, the density of microtubules extending toward the chromosomes by far exceeds the density of microtubules extending toward the cortex (for example, see Fig 1A and 8A). This difference in density correlates with the size of centriculum “holes” and the overall open area, which is significantly greater on the side of the centriculum that faces the chromosome compared to the side facing the cortex (Fig 8D and E, and Fig EV3B). If, as we suggest, the centriculum acts as a filter, then the larger its holes, the greater the density of microtubules that can pass through it, which is what we observe. We imagine two possible reasons why centriculum holes are larger on the side facing the chromosomes: first, immediately prior to metaphase, this side of the centriculum is adjacent to the pronuclei. As a result it is not as wide as other parts of the centriculum (Maheshwari et al., 2023), perhaps due to a spatial restriction of ER-derived membrane accumulation. Second, we imagine that the centriculum is a dynamic structure, much like the rest of the ER. At metaphase, microtubules that reach the chromosomes are stabilized, likely restricting the ability of the centriculum to remodel around these microtubules. Under these conditions, it is possible that additional microtubules can pass alongside the stable microtubule, further reducing the ability of the centriculum to remodel at that site and establishing a “hole” whose size may continue to increase. This explanation may also apply to cortical microtubules, which although not stabilized, may still promote the passage of additional microtubules through the centriculum alongside them. Indeed, the distribution of astral microtubules is not uniform (Fig 8A), perhaps due to the filter properties of the centriculum.

To date, the only centrosome-adjacent membrane that has been examined at nanometer resolution is the *C. elegans* centriculum. Similar membrane structures, however, have been observed in other cell types (Araújo et al., 2023; Bergman et al., 2015; Bobinnec et al., 2003; Diaz et al., 2019; Harris, 1975; Karabasheva & Smyth, 2019; Terasaki, 2000; Waterman-Storer et al., 1993). In many cases, centrosome-associated membranes are most prominent in the embryo (e.g. *C. elegans*, *Drosophila* and sea urchin). Early embryos typically contain stores of protein and mRNA that are used in the first few embryonic divisions, before the onset of zygotic expression. The centriculum and other centrosome-associated membranes may help limit the number of astral microtubules under conditions where microtubule nucleating capacity exceeds the needs of the embryo (Fig 9). Given the overall conservation in centrosome and ER-associated proteins, it is possible, if not likely, that centrosome structure and function in additional cell types are also affected by an adjacent ER-derived membrane reticulum. If this is the case, then the repertoire of proteins and processes that affect centrosome function is broader than previously appreciated, and defects in these proteins or processes might be a basis for centrosome function-related human disease.

## Materials and Methods

### Methods and Protocols

#### *C. elegans* strains maintenance

The *C. elegans* strains used in this study were derived from the N2 strain (Bristol; (Brenner, 1974) and are listed in the Reagents and Tools table. Strains were maintained at 20°C using standard methods unless noted otherwise (Brenner, 1974).

#### RNAi-Mediated Interference

##### Feeding RNAi

For *klp-7*, *tbg-1*, *zyg-9* or *smd-1* (control; (Golden et al, 2009) feeding RNAi treatments, a 5 ml Luria Broth (LB) with 50 μg/ml ampicillin were inoculated using 1:100 dilution of an overnight saturated culture (at 37°C) of *E. coli* expressing dsRNA of the gene of interest. RNAi clones were from the RNAi feeding library (Open Biosystems, Huntsville, AL) or Ahringer *C. elegans* RNAi library. Once the culture grew to OD_600_ of around 0.5 (~ 4 h at 37°C), 0.5 M IPTG (1 mM final concentration) was added to induce the bidirectional transcription of the relevant gene for another 4 h. The culture was centrifuged at 5000 g for 5 min at room temperature, and the pellet was resuspended in 1 ml of fresh LB + ampicillin (50 μg/ml) media. 200 μl of this culture were spread on each RNAi plate (MYOB with 4 mM IPTG and ampicillin 50 μg/ml). For feeding RNAi treatment, 20–40 L4-stage larvae were transferred to RNAi plates at 20°C, and after 48 h (for *klp-7*), or 24 h (for *tbg-1* and *zyg-9*), the RNAi treated worms were dissected on a glass slide, mounted on a 2% agar pad, and imaged as described below. Control RNAi treatments (*smd-1*) were done for the same amount of time as the experimental ones.

##### dsRNA injection RNAi

RNAi against *spd-5* was done using injection of double stranded RNA (dsRNA). A region of *spd-5* from 500–949 bp, was PCR amplified from N2 genomic DNA using oligonucleotides containing T7 or T3 promoter sequence. The amplified region was gel purified and then reamplified with the same PCR primers. The PCR product was purified using the Qiagen MinElute Reaction Cleanup Kit. To prepare the RNA for injection, in vitro RNA synthesis was carried out using MEGAscript^™^ T7 Transcription Kit (for forward strand) and MEGAscript^™^ T3 Transcription Kit (for the reverse strand) followed by purification using Phenol:CHCl3:Isoamyl Alcohol (25:24:1, v/v) and precipitated using 100% ethanol. The RNA pellet was dissolved in water. To prepare dsRNA, an equal amount (2 μg/μl) of both the ssRNAs were mixed and incubated at 85°C for 3 minutes in an aluminum heat block incubator followed by slow cooling to room temperature for annealing. Injection of dsRNA was done according to Ohta laboratory protocol (Ohta et al., 2021). L4s (15–20 worms) were injected with ~1 μg/μl dsRNA. These worms were maintained at 16°C for 48 h prior to live imaging of early embryos by confocal microscopy.

##### Auxin-mediated degradation

Auxin mediated degradation of ATLN-1 was done as described previously (Maheshwari et al., 2023). The *atln-1* gene was tagged with an auxin-inducible degron tag (atln-1::degron) in cells expressing TIR1, an exogenous F-box protein. Worms were transferred to bacteria seeded indole-3 acetic acid (IAA) plates (MYOB plates with 4 mM IAA) for ~20–25 minutes, and embryos were imaged immediately thereafter, as per Zhang et al 2015 (Zhang et al, 2015).

#### Confocal microscopy

##### Imaging

Images were taken using a Nikon confocal Ti2 with Yokagawa CSU-X1 spinning disk and a photometrix Prime 95B camera using a Nikon water/oil 60X 1.2-NA Apo Plan objective. Images were captured using Nikon Elements software version 5.20.00. For imaging, embryos were mounted on 2% agarose pads prepared in standard M9 buffer. Images were taken at z = 1 μm intervals unless otherwise mentioned.

##### Image analysis

All images were analyzed using Fiji (Schindelin et al., 2012), http://imagej.nih.gov/ij).

#### Measurements:

##### Centriculum diameter

Measurements were done as described in (Maheshwari et al., 2023). Two perpendicular lines (width = 7 or 1 μm) were drawn across the central focal plane of the centriculum (where the centriculum was at its largest) and through the estimated center of the centrosome, and intensity profiles of the plotted lines were obtained. A rectangle tool was used to calculate the distance between the two peaks of fluorescence intensity, resulting in two diameters (d1 and d2). The diameters were then used to determine the average diameter (d1+d2)/2 of the centriculum.

##### Centriculum void area

The inner and outer edges of the centriculum were roughly traced, creating a torodial shape that excludes the inner area (termed here the “void area”). The mean and minimum intensity values of the traced region (i.e. the torus) were determined. A (mean+min)/ 2 formula was used to determine a lower threshold value, and the Maxthr (maximum threshold, this value can also be found in measurement results where we got the mean and minimum intensity in FIJI value was used to set the upper threshold. These values were entered into the threshold function of Fiji, resulting in an accurate representation of the centriculum fluorescent signal and providing clear inner and outer edges. The inner edge of the thresholded centriculum was manually traced using a freehand selection tool in Fiji and used to define the void area by using the analyze > measure function in Fiji.

##### PCM area and PCM protein fluorescence intensity

To determine the area and total fluorescence intensity of PCM proteins (e.g. SPD-5 and TBG-1), first we determined the background mean and maximum intensity values by selecting in Fiji a region outside the PCM. The sum of mean and maximum values of the background, were used to set the lower threshold, and the Maxthr (maximum threshold) value was used to set the upper threshold, as described above. The thresholded region was selected using a wand tool. The area and total fluorescence (Raw Intensity and RawInt) were measured from the thresholded region using the analyze > measure function in Fiji. The intensity of a given fluorescently tagged protein per μm^2^ was determined by dividing the total intensity of that protein by the area that it occupies.

##### Tubulin signal intensity

For proteins that were present both at the centrosome and outside of it (e.g. TBA-1 and TBA-2, which is present in the centrosome and in the spindle), the total intensity measured here was confined to inside the centriculum. Centriculum was thresholded as described above and then the outer edge of the centriculum was traced. The image was then switched to the fluorescence channel of the protein of interest (e.g., the TBA-2 channel) and measured the intensity inside that traced region using the analyze > measure function in Fiji.

##### Peri-centrosomal microtubule area

To define the area occupied by microtubules, the microtubule signal was first roughly traced as a torus, similar to what was done for the centriculum, as described above; in all but *tbg-1* downregulation conditions, microtubules are excluded from the center of the centrosome. We then determined the mean and minimum intensity values of the traced region, as described above. A (mean+ min)/ 2 formula was used to determine the lower threshold value, and the Maxthr (maximum threshold) value was used to set the upper threshold, as described above. This resulted in the thresholded area of the pericentrosomal microtubule area. To avoid including tubulin signal from the spindle region, the spindle area was manually excluded, ensuring it is not picked up by thresholding. Finally, the outer edge of the thresholded peri-centrosomal microtubule area was traced to get the microtubule area.

##### Tubulin ring width measurement

###### Thresholding method:

The thresholding method relies on determining the areas confined by the outer and inner boundaries of the tubulin ring. The pericentrosomal microtubule area confined by the outer edge was done as described above. Assuming the area is a perfect circle, we then calculated the radius of this area, defined as radius 1 (R1). For the area confined by the inner edge, we roughly traced the void of the tubulin ring. We then determined the mean and maximum intensity values of the traced region. A (mean+ max)/ 2 formula was used to determine the lower threshold value, and the Maxthr (maximum threshold) value was used to set the upper threshold, as described above. This resulted in area confined by the inner edge of the microtubule ring (the ring “void” area), from which radius 2 (R2) was determined. Tubulin ring width was calculated by subtracting R2 from R1.

###### Line scan method:

Using Fiji, two perpendicular lines (width = 7) were drawn across the central focal plane of the tubulin ring and through the estimated center of the centrosome (avoiding the spindle region), and intensity profiles of the plotted lines were obtained, resulting in 2 peaks per profile where the line crossed the tubulin ring (4 peaks per tubulin ring). The maximum intensity of each peak was set at 100%. We then determined the x-axis coordinates at 90% of the maximum intensity of the tubulin plots, 2 coordinates for each peak (the value of 90% was chosen because the minimum value between the two peaks was typically around 85% of max intensity value). The distance between these coordinates was defined as tubulin ring width for that peak. The width of the tubulin ring was defined as the average of the four values from the 4 peaks, obtained from two line scans.

##### Microtubule angle measurement

In a zoomed-in screenshot of a 240-slice-thick section of the tomography data containing the area of interest (Maheshwari et al., 2023; Redemann et al., 2017) originally from Amira, two straight lines were drawn, marking the approximate interior and exterior edges of the centriculum (Fig 7E; while the edges of the centriculum are curved, at this resolution the edge approaches a straight line that represents the tangent line of the centriculum edge). Microtubules that crossed the interior edge were traced using the straight line tool in FIJI. If a microtubule continued beyond the exterior boundary line of the centriculum, it was categorized as a “continued MT.” Microtubules that stopped before reaching the exterior boundary were categorized as “stopped MT.” To ensure that microtubules defined as “stopped” did not continue outside the range of the slice that was being analyzed, screenshots were taken of 300 slices above and below the same area of the initial screenshot. These images were used to determine whether the traced microtubules continued in another plane. Those that did not continue on either the above or below slice set were considered stopped.

To measure microtubule angles, the angle between the inner centriculum edge line (the tangent line) and the microtubule line was determined. This angle was then subtracted from 90 degrees. The resulting value represents the angle between the microtubule and the perpendicular to the tangent line, also referred to as the normal line (Fig 7E).

### Focused-Ion beam scanning electron microscope (FIB-SEM) imaging

The FIB-SEM data used in this study was previously published (Rahman et al, 2020). 3D segmentation of centriculum structure were done as previously published (Rahman et al., 2020) using Amira 6 (version 3D 2022.2, FEI/Thermo Fisher Scientific) default general scheme to segment selected ROI in a semi-autonomous method. Briefly, first, we selected centriculum membranes using a threshold followed by at least three rounds of manual inspection (slice by slice through X, Y, and Z separate planes) to remove incorrect segments and add unsegmented membranes to the final membrane volume (Maheshwari et al., 2023; Rahman et al., 2020).

To measure the size of centriculum openings (namely open space between reticular membranes that extend all the way through the centriculum), we placed fiducials (on the same plane) next membrane edges on the pronuclear and cortical size of the centriculum. We took TIFF screenshots of the centriculum membrane from behind a central fiducial (i.e., equal distance from the pronuclear and cortical centriculum membrane fiducials) placed on the same plane as perinuclear/cortical fiducials. For each TIFF image, we determined a scale bar separately by marking 11 consecutive pixels as 100 nm. We measured the size of each hole using the threshold-based selection and area measurement tool in Fiji (version 2.14.0/1.54f). To ensure that the centriculum segments in these images are *en face*, we restricted the angle between the center plane and the top and bottom of each image to less than 30°. The actual angle was determined by drawing straight lines from the central fiducial to fiducials that were placed on the top bottom edges of each image. Next, we took TIFF images of the perpendicular view of the above-mentioned lines and utilized the angle measurement tool in Fiji.

### Quantification and statistical analysis

#### Statistical analysis

All analyses were done using GraphPad Prism. For a normally distributed sample, a unpaired t-test was used; otherwise, a two-tailed Mann-Whitney test was used as a non-parametric test. For comparison between multiple datasets, one-way ANOVA non-parametric Kruskal-Wallis test with correction for multiple comparisons was used. The statistical tests used, the number of samples analyzed, and the p-values are indicated in the figure legends. The criterion for statistical significance was set at p < 0.05. Results are represented as mean ± standard deviation unless indicated otherwise. The binned frequency distribution graphs in Fig 7, 8 and EV3 was done using Microsoft excel. The bin width was set at 500 nm^2^ up to 1000 nm^2^ then from 1001 to 10000 the data were binned at every 1000 nm^2^ with overflow bin set at >10000 nm^2^.

## Supplementary Material

Supplement 1

## Figures and Tables

**Reagents and Tools table:** 

Reagent/Resource	Reference or Source	Identifier or Catalog Number
		
**Experimental Models**		
Bacterial strains		
Escherichia coli clone for *C. elegans* SMD-1 F47G4.7	RNAi feeding library (Open Biosystems, Huntsville, AL)	N/A
Escherichia coli clone for *C. elegans* KLP-7 K11D9.1	RNAi feeding library (Open Biosystems, Huntsville, AL)	N/A
Escherichia coli clone for *C. elegans* ZYG-9 F22B5.7	RNAi feeding library (Ahringer RNAi library)	N/A
Escherichia coli clone for *C. elegans* TBG-1 F58A4.8	RNAi feeding library (Open Biosystems, Huntsville, AL)	N/A
***C. elegans* strains**		
*C. elegans* wild type isolate	CGC	N2
unc-119(ed3) III; ltIs76 [pAA178: pie-1p::mCHERRY::sp12 + unc-119 (+)]; ltIs25 [pAZ132; pie-1p::GFP::tba-2 + unc-119 (+)]	Audhya lab	MSN146
*ebp-2(or1954[ebp-2::mKate2]) II; mTurquoise2::H2B (his-72::linker::mTurquoise2) III (CRISPR); unc-119(ed3); ojIs23 [SP12::GFP + unc-119(+)]*	(Maheshwari et al., 2023)	OCF163
*spd-5(vie26[gfp::spd-5 +loxP]) I; unc-119(ed3); ocfIs2[pie-1 prom::mCherry::SP12::pie-1 3’UTR +unc119 (+)];mTurquoise2::H2B (his-72::linker::mTurquoise2) III*	(Maheshwari et al., 2023)	OCF176
*unc-119(ed3); ocfIs2[pie-1 prom::mCherry::SPl2::pie-1 3’UTR +unc119 (+)];ltIs25 [pAZ132; pie-1p::GFP::tba-2 + unc-119 (+)]; mTurquoise2::H2B (his-72::linker::mTurquoise2) III*	(Maheshwari et al., 2023)	OCF181
*unc-119(ed3); ocfIs2[pie-1 prom::mCherry::SP12::pie-1 3’UTR +unc119 (+)];ltIs25 [pAZ132; pie-1p::GFP::tba-2 + unc-119 (+)]; mTurquoise2::H2B (his-72::linker::mTurquoise2) III; ocf101[atln-1::3xFLAG::degron] IV (CRISPR); TIR1::mRuby IV*	(Maheshwari et al., 2023)	OCF183
*unc-119(ed3); ojIs23 [SP12::GFP + unc-119(+)]; itSi569Itbg-1::mCherry); mTurquoise2::H2B (his-72::linker::mTurquoise2) III*	(Maheshwari et al., 2023)	OCF184
*unc-119(ed3); ocfIs2[pie-1 prom::mCherry::SP12::pie-1 3’UTR +unc119 (+)]; cb-unc-119(+)]I; ltSi1141[pOD1021/pVV103; Pspd-2::GFP::SPD-5 reencoded; cb-unc-119(+)]II; unc-119(ed3) III*	This study	OCF187
*unc-119(ed3); ocfIs2[pie-1 prom::mCherry::SP12::pie-1 3’UTR +unc119 (+)]; ltSi592[pVV168; Pspd-2::GFP-spd-5 mut 653,658, reencoded; cb-unc-119(+)]II; unc-119(ed3)III*	This study	OCF189
*unc-119(ed3); ojIs23 [SP12::GFP + unc-119(+)]; PH::gfp; tba-1(pg77[tba-1::TagRFP-T + loxP]) I; uIs31 III*	This study	OCF193
*ltSi592[pVV168; Pspd-2::GFP::spd-5 mut 653,658, reencoded; cb-unc-119(+)]II; unc-119(ed3)III; spd-5(wow36[tagrfp-t^3xmyc::spd-5]) I*	This study	OCF200
*Pspd-2::GFP::SPD-5 reencoded; cb-unc-119(+)]II; unc-119(ed3) III; spd-5(wow36[tagrfp-t^3xmyc::spd-5]) I*	This study	OCF201
*tba-1(pg77[tba-1::TagRFP-T + loxP]) I; uIs31 III; Pspd-2::GFP::SPD-5 reencoded; cb-unc-119(+)]II; unc-119(ed3) III*	This study	OCF212
*tba-1(pg77[tba-1::TagRFP-T + loxP]) I; uIs31 III; ltSi592[pVV168; Pspd-2::GFP::spd-5 mut 653,658, reencoded; cb-unc-119(+)]II; unc-119(ed3)III*	This study	OCF213
*unc-119(ed3); ojIs23 [SP12::GFP + unc-119(+)];tba-1(pg77[tba-1::TagRFP-T + loxP]) I; ltSi1219[pMO104; Pspd-2::SPD-5 (re-encoded) (WT, untagged);cb-unc-119(+)]II; unc-119(ed3)III*	This study	OCF214
*unc-119(ed3); ojIs23 [SP12::GFP + unc-119(+)]; tba-1(pg77[tba-1::TagRFP-T + loxP]) I; ItSi1219[pMO104; Pspd-2::spd-5 S653A S658A::spd-5 3′UTR; cb-unc119(+)]II; unc-119(ed3)III*	This study	OCF215
**Recombinant DNA**		
Vector for expressing dsRNA: pL4440	Addgene	# 1654
**Antibodies**		
None		
**Oligonucleotides and other sequence-based reagents**		
TAATACGACT CACTATAGGT GGAATT GTCCGCTACTGAT	IDT	*spd-5* RNAi F
AATTAACCCT CACTAAAGGT GTATT C AACGAGTGCCTGA	IDT	*spd-5* RNAi R
**Chemicals, Enzymes and other reagents**		
Amplicillin	Sigma Aldrich	Cat # A9518
Indole-3-acetic acid (auxin)	Alfa Aesar	Cat # A10556
IPTG	Sigma Aldrich	Cat # I6758
Qiagen MinElute Reaction Cleanup Kit Qiagen Cat	Qiagen	Cat # 28206
MEGAscript^™^ T7 Transcription Kit	Invitrogen	Cat # AM1333
MEGAscript^™^ T3 Transcription Kit	Invitrogen	Cat # AM1338
Phenol:CHCl3:Isoamyl Alcohol	Invitrogen	Cat # 15593031
Ethanol 200 proof	The Warner-Graham company	Cat # 64-17-5
Agarose	Invitrogen	Cat # 214010
**Software**		
FIJI (ImageJ release 2.1.0)	(Schindelin et al, 2012)	https://imagej.nih.gov/ij/
GraphPad Prism [Version 10.1.1 (270)]	GraphPad	https://www.graphpad.com/scientificsoftware/prism/
Nikon Elements Software	Nikon USA	N/A
IMOD or 3dmod [version 4.11.1]	https://bio3d.colorado.edu	N/A
AMIRA 6.5.0 (release 2018-03-07)	Thermo Fisher Scientific	N/A
Amira ZIB edition Version 2020.1	Zuse Institute Berlin Dep. Visual Data Analysis, Takustr.7, Berlin, Germany.	N/A
Adobe Photoshop CC (release 23.1)	Adobe	N/A
**Other**		
		
		
