# Supplementary material for: The centriculum, a membrane that surrounds C. elegans centrosomes, acts as a microtubule filter
Source: bioRxiv. 2025 Aug 18:2025.08.16.670680. Preprint. [Version 1] doi: 10.1101/2025.08.16.670680 (PMC12393313; doi:10.1101/2025.08.16.670680)
Supplement: Supplement 1 [file media-1.pdf]

Extended view Figures and Legends

Extended View Figure EV1

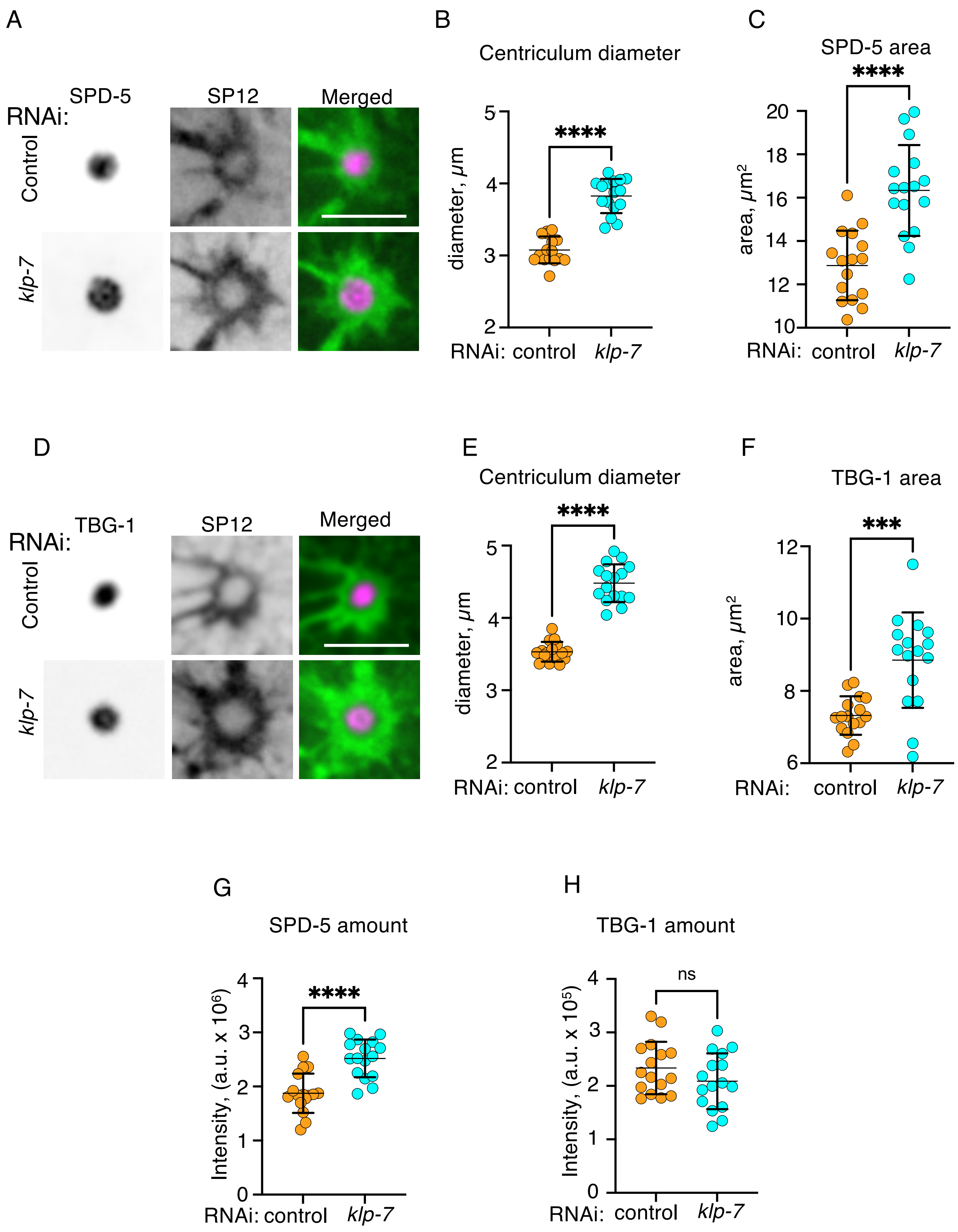

Figure EV1: Microtubule treatments that affect centriculum size also affect PCM size

A) Representative images of centricula from 1-cell embryos (OCF176) at metaphase expressing mCherry::SP12 (green in merged image) and GFP::SPD-5 (magenta in merged image), following control or *klp-7* RNAi. Scale bar= 5 µm.

B,C and G) Quantification of average centriculum diameter, SPD-5 area and SPD-5 amount from embryos treated as shown in A. n=16 for control (orange) and *klp-7* (cyan) RNAi treatment. $p<0.0001$ for graphs using unpaired t test. Error bars represent mean and standard deviation.

D) Representative images of centricula from 1-cell embryos (OCF184) at metaphase expressing SP12::GFP (green in merged image) and TBG-1::RFP (magenta in merged image), following control or *klp-7* RNAi treatment.

E, F and H) Quantification of average centriculum diameter and the area occupied by TBG-1 from embryos treated as shown in panel D. n=16 for control (orange) and *klp-7* (cyan) RNAi. $p<0.0001$ (panel E), $p=0.0002$ (panel F) and $p=0.1781$ (panel H) using unpaired t test. Error bars indicate mean and standard deviation.

Extended View Figure EV2

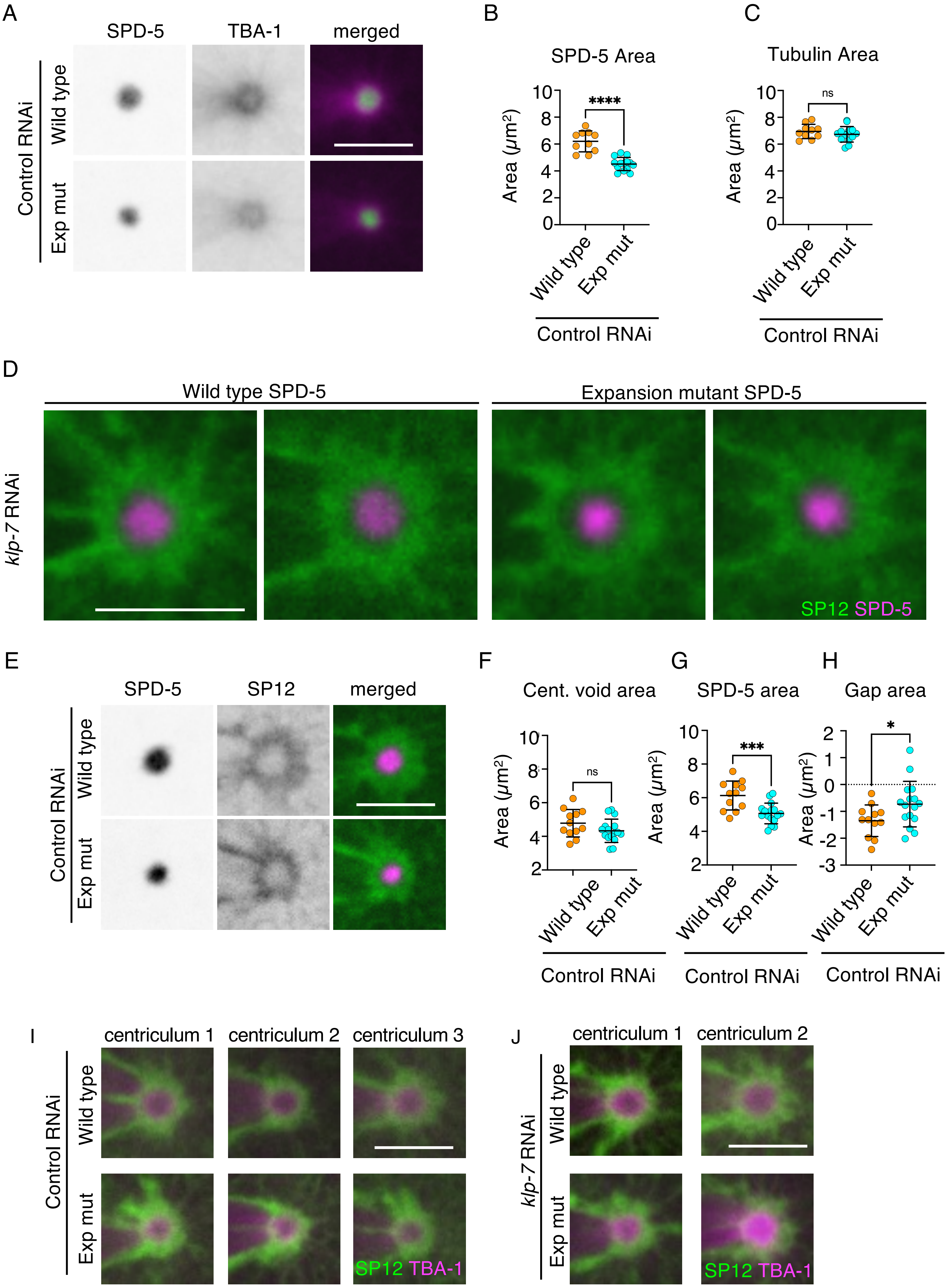

Figure EV2: The relationship between the centriculum, microtubules and the PCM in the presence of the SPD-5 expansion mutant.

A) Representative images of centricula from 1-cell embryos at metaphase expressing endogenous TBA-1::RFP and either transgenic GFP::SPD-5 (OCF212) or transgenic GFP::SPD-5^{exp} (OCF213) after control RNAi treatment. Images for the *klp-7* RNAi condition are shown in Fig 3I. Scale bar = 5 µm.

B and C) Quantification of SPD-5 area and TBA-1 area from the embryos treated as shown in panel A. n=10 and n=14 for the transgenic wild type (orange) and expansion mutant SPD-5 (cyan), respectively. $p<0.0001$ (panel B) and $p= 0.3707$ (panel C) using the unpaired t test. Error bars indicate mean and standard deviation.

D) Additional images to the one shown in Fig 6C of centricula using mCherry::SP12 (green in merged images) and either transgenic wild type GFP::SPD-5 (magenta in merged images; strain OCF187) or transgenic GFP::SPD-5^{exp} (magenta in merged images; strain OCF189) following *klp-7* RNAi treatment. Note the gap between the PCM (as seen using SPD-5) and the centriculum. Scale bar = 5 µm.

E) Representative images of the same experiment in panel D, but with control RNAi treatment. Scale bar = 5 µm.

F-H) Quantification of centriculum void area, SPD-5 area, and the gap area from embryos treated as shown in panel E. p values were 0.1278 (panel F), 0.0007 (panel G) and 0.0404 (panel H), using unpaired t test. n=12 and 16 for transgenic wild type (orange) and SPD-5 exp mutant, (cyan) respectively. Quantification of the same parameters under *klp-7* RNAi conditions is shown in Fig 6D-F.

I-J) Images from control conditions of centricula and peri-centrosomal microtubules from 1-cell embryos expressing SP12::GFP (green in merged image) and TBA-1::RFP (magenta in merged image) and either transgenic wild type SPD-5 (OCF214) or SPD-5^{exp} (OCF215) and additional images to the one shown in Fig 6G following *klp-7* RNAi treatment. Scale bar = 5 µm.

Extended View Figure EV3

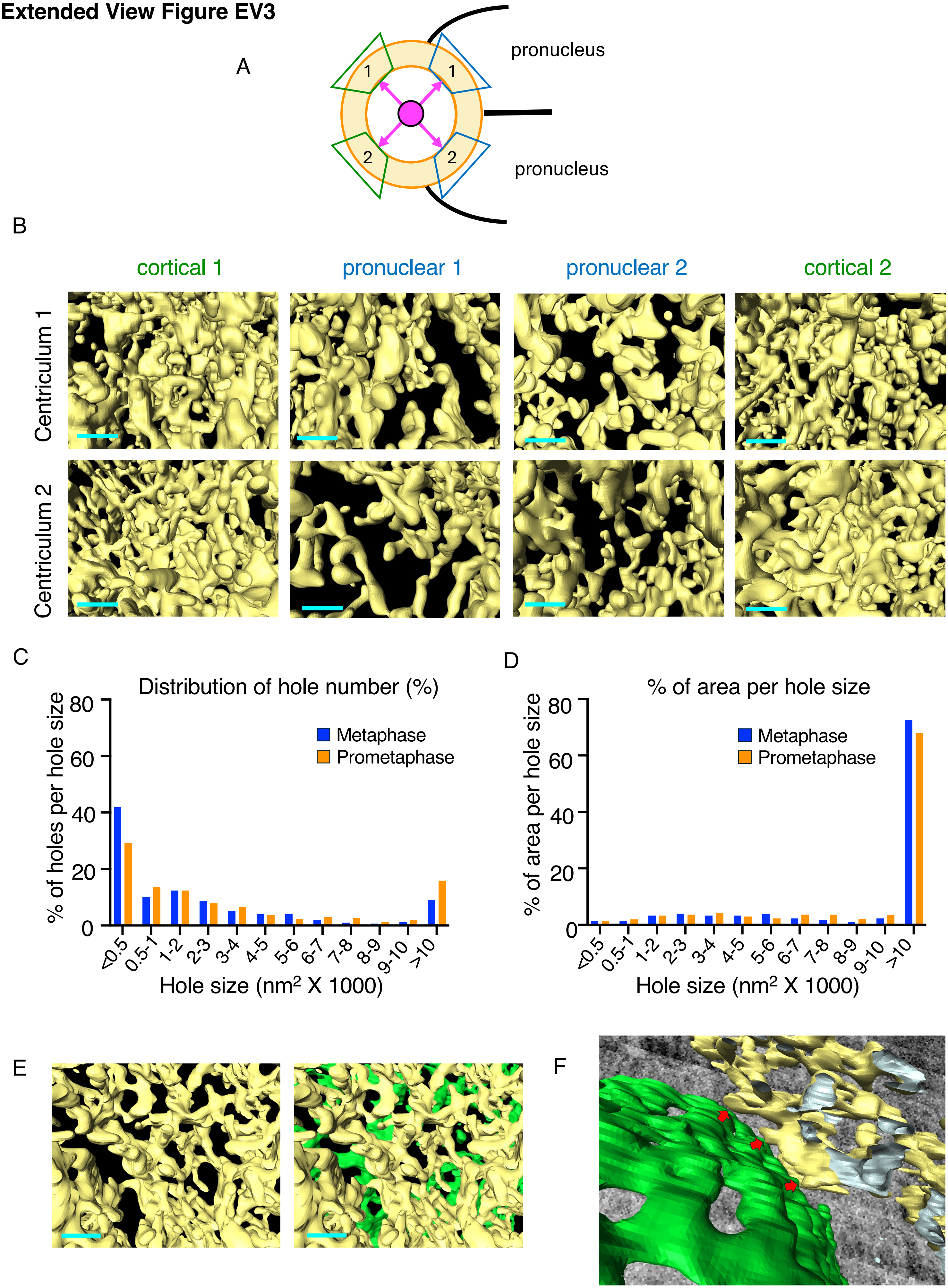

Figure EV3: Centriculum porosity is greater on the side facing the pronuclei compared to the side facing the cortex.

A) Diagram depicting the centriculum (in yellow) fused to the two pronuclei (black lines) at a metaphase 1-cell embryo. The areas highlighted are on the cortical side (green trapezoids) and pronuclear side (blue trapezoids), which are shown in panel B. The numbers refer to the order of the images shown below. A central fiducial (in pink) is equidistance (pink arrows) from the areas from which the images were taken.

B) Cortical and pronuclear centriculum segments based on FIB-SEM data (Maheshwari *et al.*, 2023) from two centricula, shown side by side. The images were taken from the central fiducial, as described in Fig 7A and 8B. The order of the images is as indicated in panel A. Scale bar= 200 nm.

C) Binned frequency distribution of holes present on the pronuclear side of metaphase (in blue) and prometaphase (orange) centricula from a 1-cell embryos. The metaphase data are the same as shown in Fig 8D. Bin size range is shown on the X axis. n=308 for prometaphase holes from 6 images, taken from 3 centricula.

D) Binned frequency distribution of the percentage of total open area per hole size range, for holes in metaphase (in blue) and prometaphase (orange) centricula from a 1-cell embryos, using the same data as in panel C. The data for holes on the cortical size are the same as shown in Fig 8E.

E) A second example of the pronuclear side of a prometaphase centriculum (in yellow), without (left side) and with (right side) segmentation of nuclear membrane remnants (in green). Based on FIB-SEM data (Maheshwari *et al.*, 2023). Areas were visualized from the center of the centrosome. Scale bar= 200 nm.

F) Areas of possible contact (indicated by red arrows) between a prometaphase centriculum (in yellow) and remnants of the nuclear membrane (in green) based on FIB-SEM data (Maheshwari *et al.*, 2023). In this image, the centrosome is to the upper right side while the chromosomes are to the lower left side.
